# *nMNSD*—A Spiking Neuron-Based Classifier That Combines Weight-Adjustment and Delay-Shift

**DOI:** 10.3389/fnins.2021.582608

**Published:** 2021-02-19

**Authors:** Gianluca Susi, Luis F. Antón-Toro, Fernando Maestú, Ernesto Pereda, Claudio Mirasso

**Affiliations:** ^1^UPM-UCM Laboratory of Cognitive and Computational Neuroscience, Centro de Tecnologia Biomedica, Madrid, Spain; ^2^Departamento de Psicología Experimental, Facultad de Psicología, Universidad Complutense de Madrid, Madrid, Spain; ^3^Department of Civil Engineering and Computer Science, University of Rome “Tor Vergata”, Rome, Italy; ^4^CIBER-BBN: Networking Research Center on Bioengineering, Biomaterials and Nanomedicine, Madrid, Spain; ^5^Departamento de Ingeniería Industrial & IUNE & ITB. Universidad de La Laguna, Tenerife, Spain; ^6^Instituto de Física Interdisciplinar y Sistemas Complejos (IFISC, UIB-CSIC), Palma de Mallorca, Spain

**Keywords:** classification, delay learning, MNSD, online learning, spike latency, heterosynaptic plasticity, MNIST database

## Abstract

The recent “multi-neuronal spike sequence detector” (MNSD) architecture integrates the weight- and delay-adjustment methods by combining heterosynaptic plasticity with the neurocomputational feature spike latency, representing a new opportunity to understand the mechanisms underlying biological learning. Unfortunately, the range of problems to which this topology can be applied is limited because of the low cardinality of the parallel spike trains that it can process, and the lack of a visualization mechanism to understand its internal operation. We present here the nMNSD structure, which is a generalization of the MNSD to any number of inputs. The mathematical framework of the structure is introduced, together with the “trapezoid method,” that is a reduced method to analyze the recognition mechanism operated by the nMNSD in response to a specific input parallel spike train. We apply the nMNSD to a classification problem previously faced with the classical MNSD from the same authors, showing the new possibilities the nMNSD opens, with associated improvement in classification performances. Finally, we benchmark the nMNSD on the classification of static inputs (MNIST database) obtaining state-of-the-art accuracies together with advantageous aspects in terms of time- and energy-efficiency if compared to similar classification methods.

## 1. Introduction

In the last few years, diverse machine learning (ML) methods have been proposed for the recognition of spike patterns generated by neural populations (Ambard and Rotter, 2012; Tapson et al., 2013; Grassia et al., 2017; Nazari and Faes, 2019). The ability to learn and decode spike patterns is not only useful for the interpretation of biological mechanisms (Koyama et al., 2010; Rudnicki et al., 2012; Heelan et al., 2019) but also for engineering applications, such as artificial vision and hearing (Nogueira et al., 2007; Zai et al., 2015; Schofield et al., 2018) analysis of brain signals (Susi et al., 2018), forecasting of energy consumption (Kulkarni et al., 2013), and so on. Most of such ML methods are based on neural networks, and specifically on the bio-inspired spiking neural networks (SNNs) (Maass, 1997; Florian, 2012).

In the literature, there are many learning methods for SNNs that make use of biologically plausible strategies. While most of these methods are based on synaptic learning rules aimed at modifying the weights (i.e., *weight adjustment*), only few of them consider the modulation of the delay time to achieve learning (i.e., *delay shift*, see Brückmann et al., 2004; Adibi et al., 2005; Taherkhani et al., 2015; Matsubara, 2017; Hwu et al., 2018; Wang et al., 2019). Interestingly, it has been demonstrated that the alteration of delays has advantages in forming spatiotemporal memories, over altering synaptic weights (Izhikevich, 2006; Hwu et al., 2018). Incidentally, experimental research proved that delays are widely present in biological neural networks and contribute to encode information (Chase and Young, 2007; Minneci et al., 2012), and various biological justifications have been attributed to the delay adjustment processes, among which the activity-dependent myelination (Mount and Monje, 2017) (which, in turn, results in the modulation of conduction velocities) and the spike latency tuning (see Fields, 2008, 2015; Zhou et al., 2012; Matsubara, 2017; Hwu et al., 2018; Wang et al., 2019).

The recently developed multi-neuronal spike sequence detector (MNSD) architecture (Susi et al., 2018) is a simple but effective bio-inspired topology specialized in online learning and recognition of parallel spike trains. Such tool integrates the weight- and delay-adjustment methods by means of the spike timing-dependent plasticity (STDP) and the well-known mechanism of spike latency (i.e., the neuron's intrinsic potential-dependent delay time between the overcoming of the “threshold” and the actual spike generation, Izhikevich, 2004; Salerno et al., 2011), representing a new opportunity to understand the mechanisms underlying biological learning.

**Figure 1:**
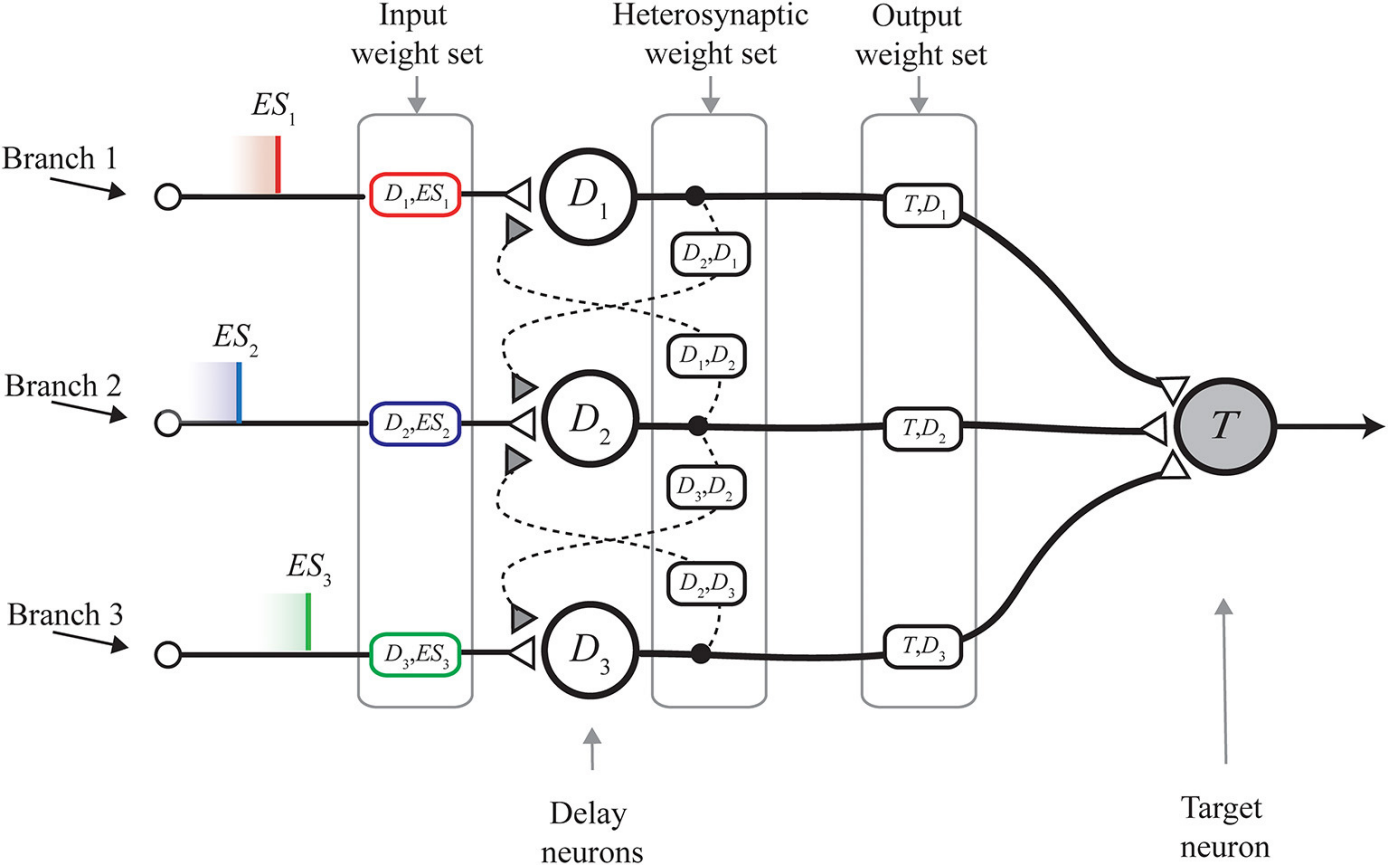
Multi-neuronal spike sequence detector (MNSD) structure hit by the parallel spike train *ES*_1_, *ES*_2_, *ES*_3_. The different sections of the structure are shown. From **left** to **right**: input weight set, heterosynaptic weight set, output weight set; delay and target neuron are indicated with *D*_*i*_ and *T*, respectively. Modified from Susi et al. (2018).

In its original form, the MNSD architecture (Figure 1) is composed of:

- A layer of *delay neurons* *D*_1_, *D*_2_, *D*_3_ (termed delay layer), which receive the parallel spike train (composed of the external spikes *ES*_1_, *ES*_2_, *ES*_3_). Such neurons are characterized by nearest-neighbor excitatory interactions mediated by heterosynaptic STDP (the dashed links in Figure 1); this mechanism allows the synaptic weights of the neurons to change on the basis of the current parallel spike train. Through the spike latency feature, the change in the weight reflects on the modulation of the delay in relaying the external spike received on the *i-th* branch, toward next stages of the structure. In this way, the MNSD is able to learn the input parallel spike train, which can be represented into a multi-dimensional, temporal, feature space (Susi et al., 2018);
- One *target neuron* *T*, which performs the summation of the outputs of the three delay neurons and acts as readout neuron, signaling the recognition of a specific parallel spike train. In order for the target to produce a spike, a synchrony of the contributions arriving from previous steps of the structure is required. It happens only if the delays introduced by the *delay neurons* are able to compensate the initial lags among the spikes of the input parallel spike train;
- Three sets of weights, each one for a family of connections. The *input weight set* (i.e., the set of variables 〈*w*__*D*_1_, *ES*_1___, *w*__*D*_2_, *ES*_2___, ..., *w*__*D*_*n*_, *ES*_*n*___〉, each one representing an *input weight*), that is where the learning is finally encoded; the input weights, that are subject to the action of STDP, are placed between the input terminals and the delay layer. The *heterosynaptic weight set* (i.e., the set of variables 〈*w*__*D*_1_, *D*_2___, *w*__*D*_2_, *D*_1___, *w*__*D*_2_, *D*_3___, *w*__*D*_3_, *D*_2___, ..., *w*__*D*_*n*−1_, *D*_*n*___, *w*__*D*_*n*_, *D*_*n*−1___〉 each one representing a *heterosynaptic weight*); the heterosynaptic weights, which are placed between adjacent delay neurons, are characterized by a very low value and serve for the STDP-based adjustment mechanism of the input weights. The *output weight set* (i.e., the set of variables 〈*w*_*T*,*D*_1__, *w*_*T*,*D*_2__, ..., *w*_*T*,*D*_*n*__〉, each one representing an *output weight*); the output weights are placed between the delay layer and the target neuron, and allow us to control the target summation. This is done by assigning the relevance of each feature in the definition of the pertaining class. In other words, the output weight set establishes the shape of the delimiter of a class in the feature space.

The described structure is able to perform online learning and recognition. Obviously, it can also be used envisaging separately a learning phase (with STDP activated) and a recognition phase (with STDP disabled).

Unfortunately, the range of problems to which the original version of the MNSD can be applied is limited because of two reasons: the low cardinality of the parallel spike trains that can be processed (3 branches, i.e., 3 features per class) and the lack of a tool able to represent the internal computation in order to set the parameters in accord to the specification of the problem in a knowingly manner.

**Figure 2:**
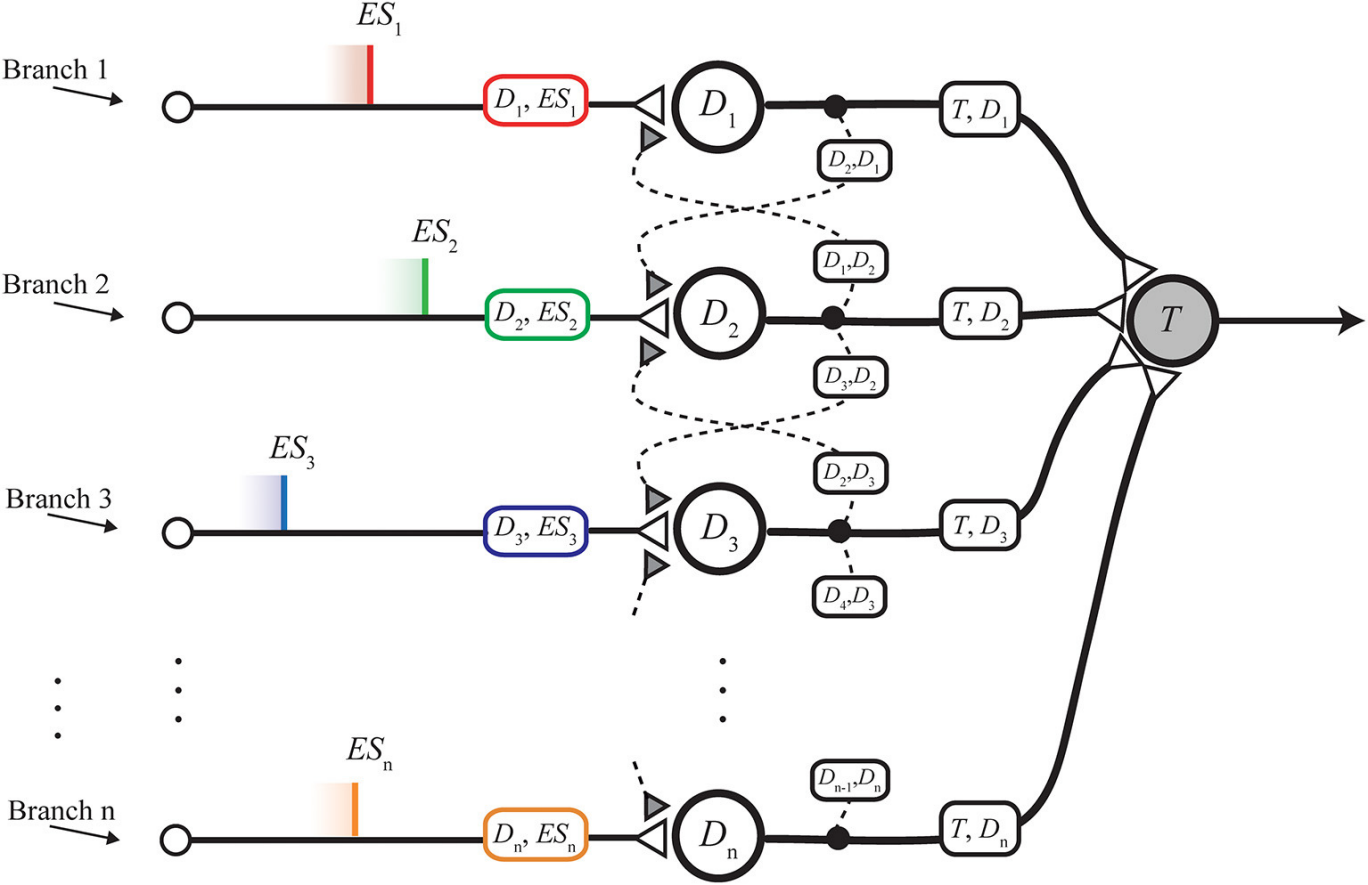
nMNSD hit by a parallel spike train.

In this work, we present various novelties:

- The nMNSD structure *per se* (Figure 2), which is a generalization of the MNSD to any number of inputs. The analysis of the internal operation of the structure is presented, considering the two operating modalities highlighted in Susi et al. (2018):
  – *Static behavior*, i.e., how an nMNSD with a specific weight configuration will react to a new given input sequence (disregarding the action of STPD);
  – *Dynamic behavior*, i.e., how an nMNSD with a specific set of STDP parameters will change its input weights in consequence to a new given input sequence.
  To improve the practical usability of the nMNSD as a classifier tool, we will discuss two possibilities: (1) to regulate the impact of each feature in the target summation for the determination of the class (i.e., *feature relevance* property), and (2) to configure different nMNSDs to be used in a multiclass problem where the number of branches is the number of features, and the number of nMNSD structures used is the number of classes.
- The *trapezoid method*, i.e., a reduced method to analyze the recognition mechanism operated by the nMNSD in response to a specific input sequence. This serves as design support regarding the static behavior of the nMNSD, and results necessary to represent its internal processing with a number of branches greater than 3, since in this case the feature space is not trivially representable (more than 3 dimensions). We provide a visualization toolbox based on this method, which allows the user to consciously customize nMNSD-based classification systems for specific classification problems.
  Finally, we present 2 applications of the nMNSD. We apply our extended method to a classification problem previously faced with the classical MNSD from the same authors, showing the new possibilities the nMNSD opens, with associated improvement in classification performances. Finally, we benchmark the nMNSD on the classification of handwritten digits from the MNIST database, obtaining state of the art accuracies together with advantageous aspects in terms of time- and energy-efficiency if compared to similar classification methods.

The visualization toolbox, based on a dedicated nomogram (see Appendix 1 in Supplementary Material), can be found at the following weblink: www.github.com/LCCN/Frontiers2021.

## 2. Materials and Methods

### 2.1. A Brief Resume on the LIFL Neuron Model

The LIFL neuron model (Cardarilli et al., 2013; Susi et al., 2018) is similar to the classical Leaky Integrate-and-Fire (LIF), but it is characterized by the presence of the *spike latency* neurocomputational feature (Izhikevich, 2004; Salerno et al., 2011), a real neuron characteristic that has been extracted from the Hodgkin–Huxley equations (Salerno et al., 2011). In a nutshell, it consists of the neuron's intrinsic potential-dependent delay time between the overcoming of the “threshold” and the actual spike generation, allowing the neuron to encode the strength of the input in the spike times. The LIFL neuron model is characterized by an internal state *S* that represents the membrane potential of the biological counterpart. *S* conventionally ranges from 0 (representing the resting value, *S*_0_) to a maximum value *S*_*max*_ (at most ∞), and a fixed threshold *S*_*th*_, slightly greater than 1. The value of *S* with respect to *S*_*th*_ demarcates two different working modes of the neuron:

- the *passive mode*, for *S* < *S*_*th*_. Here, the evolution of *S* during time is characterized by a spontaneous decay. Although the equations of the LIFL neuron are compatible with different decay types, we will consider here for simplicity a linear subthreshold decay (asin Susi et al., 2018; Mattia and Del Giudice, 2000). Accordingly, given a temporal distance Δ*t* between two consecutive incoming spikes, *S* experiences a decrease, such that:
  (1) $$\begin{array}{l} S_{new}=S_{old}-L_{d}\cdot \Delta t \end{array}$$
  being *L*_*d*_ a non-negative quantity called *decay parameter*.
- the *active mode*, for *S* ≥ *S*_*th*_. Pnce *S* crosses the value *S*_*th*_, the neuron is ready to fire; however, firing is not instantaneous, but it occurs after a continuous-time delay, the model equivalent of the spike latency feature of real neuron, that we call *time-to-fire* and indicate with *ttf*:

(2) $$\begin{array}{l} ttf=\frac{1}{S-1} \end{array}$$

The latter defines the relationship between *S* and *ttf*.

Here, the evolution of *S* is characterized by a spontaneous growth:

(3) $$\begin{array}{l} S_{new}=\frac{{\left(S_{old}-1\right)}^{2}\Delta t}{1-\left(S_{old}-1\right)\Delta t} \end{array}$$

Obviously, in the case of a transition from passive to active mode, Equation (1) is applied (Equation 3 if vice versa, although we will not consider this case since inhibitory contributions are not envisaged in this work).

The firing threshold is written as:

(4) $$\begin{array}{l} S_{th}=1+d \end{array}$$

where *d* is a positive value called *threshold constant*, which fixes a bound for the maximum value of *ttf*. According to Equation (2), when *S* = *S*_*th*_, *ttf* is maximum, and equals to:

(5) $$\begin{array}{l} ttf_{max}=\frac{1}{d} \end{array}$$

*ttf*_*max*_ represents the upper bound of the time-to-fire and is a measure of the finite maximum spike latency of the biological counterpart (FitzHugh, 1955).

Simple Dirac delta functions (representing the action potentials) are exchanged between neurons in form of pulses or pulse trains.

### 2.2. Pattern Recognition in a Trained nMNSD

The nMNSD is an extension of the MNSD to an arbitrary number of branches (Figure 2). This gives the possibility to face classification problems characterized by an arbitrary number of features, *n*.

We give in this section a summary of the static behavior of an nMNSD structure (i.e., as multi-neuronal spike pattern detector, disregarding the action of the STDP) and evaluate how the structure will react with regard to a specific input parallel pattern, in accord to the structure's input weight set.

**Figure 3:**
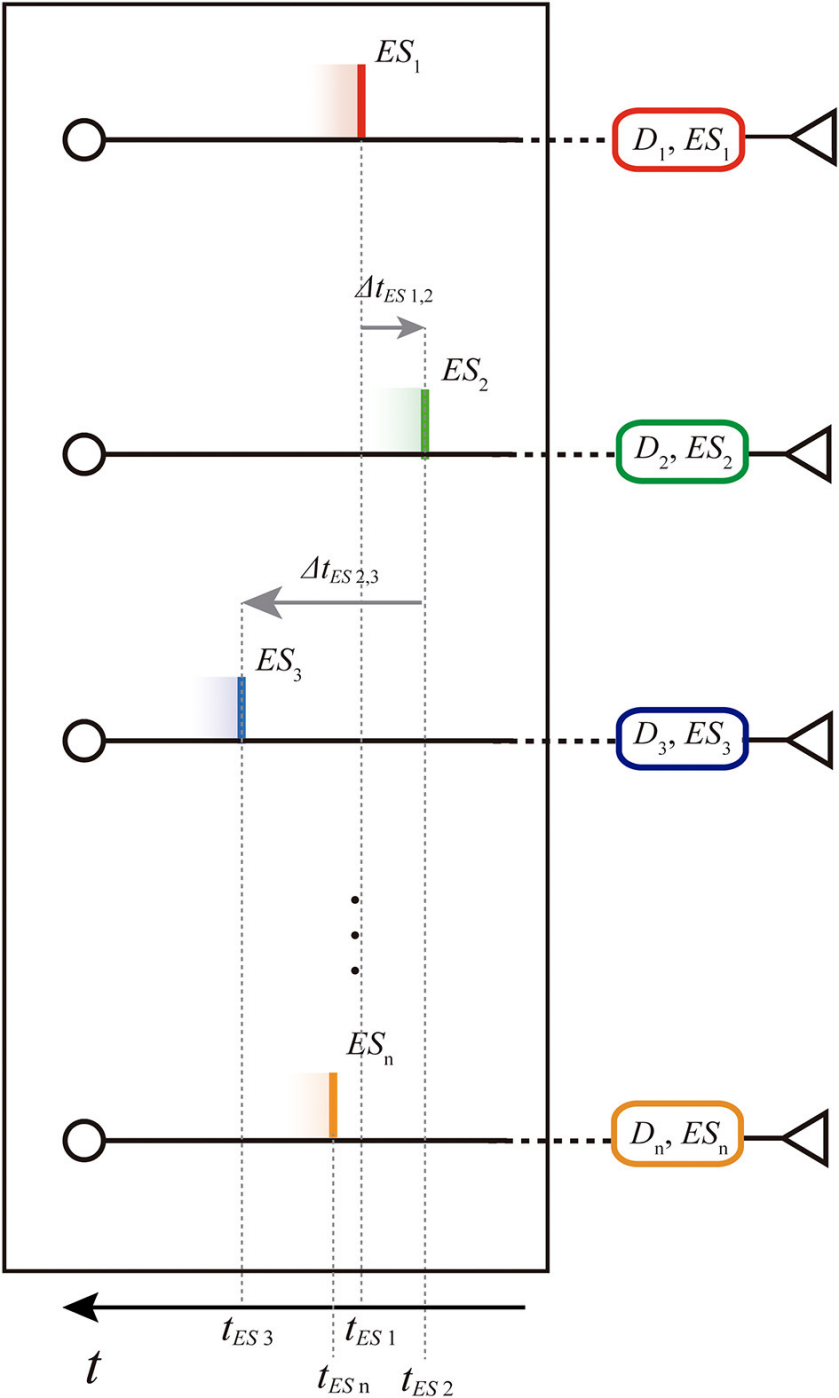
Representation of the spike train shown in Figure 2 on the entryway of the structure: absolute spike times (*t*_*ES*1_, *t*_*ES*2_,., *t*_*ESn*_) and relative intervals (Δ*t*_*ES*1, 2_, Δ*t*_*ES*2, 3_, ..., Δ*t*_*ESn*−1, *n*_). Note that the time axis is reversed since we are representing the parallel spike train in motion toward right.

Assumed the same amplitude *A*_*ES*_*i*__ = 1 for each single external input spike *ES*_*i*_ (see Table 1), a parallel spike train of order *n* is characterized by a vector of *n* external *absolute spike times* of consecutive branches 〈*t*_*ES*_1__, *t*_*ES*_2__, ..., *t*_*ES*_*i*__, ..., *t*_*ES*_*n*__〉 (positive values), one for each component spike. In order to make our procedure independent of the initial time offset, and for ease representation, in some parts of this document the parallel spike train can be equivalently defined by the vector of *n* − 1 intervals between spike events of consecutive branches, i.e., *relative intervals* 〈Δ*t*_*ES*_1, 2__, Δ*t*_*ES*_2, 3__, ..., Δ*t*_*ES*_*n*−1, *n*__〉 (which components can assume positive or negative values) (see Figure 3).

**Table 1:** Recommended values for the nMNSD structure parameters (see the reference paper Susi et al., 2018).

**Parameter**	**Value / Condition**
Neuron parameters	Identical for all neurons:
	*S*_0_ = 0
	*L*_*d*_ ≤ 0.15
	*S*_*th*_ = 1.04 (^*^)
ES amplitudes	*A*_*E*_*S*__*i*__ = 1.00 (^**^)
Input weights (starting value)	*w*_*D*_*i*_, *ES*_*i*__≃1.08 (^***^)
Target weights	$\sum_{i=1}^{n}w_{{}_{T,D_{i}}}\geq S_{th}$
STDP parameters	*A*_+_ = −*A*_−_ ≤ 0.01; τ+ = τ_−_≃[2−10]
Heterosynaptic weights	*w*_*D*_*i*_, *D*_*i*_+1_ = *w*_*D*_*i*+1_, *D*_1__≃0 (^****^)
Connection delays	0 (instantaneous)

(*) *Able to ensure a value of *ttf*_*max*_ sufficiently high to differentiate the input patterns (*ttf*_*max*_*D*__*i*__ = 25 ms)*.

(**) *Giving the same weight for all the input spikes is a simplifying but not unrealistic assumption, since spike amplitude has been observed to change mostly as function of the firing rate of the spiking neuron's activity (Stratton et al., 2012), which in our experiments can be considered very low and quite constant*.

(***) *Chosen to let *D*_*i*_ generate a spike around the center of the latency range (i.e., *ttf*(1.08) = *ttf*_*max*_*D*__*i*__/2 = 12.5 ms), then obtain a large variation margin for the weight adjustments during learning*.

(****) *Lateral contributions are considered weak*.

**Figure 4:**
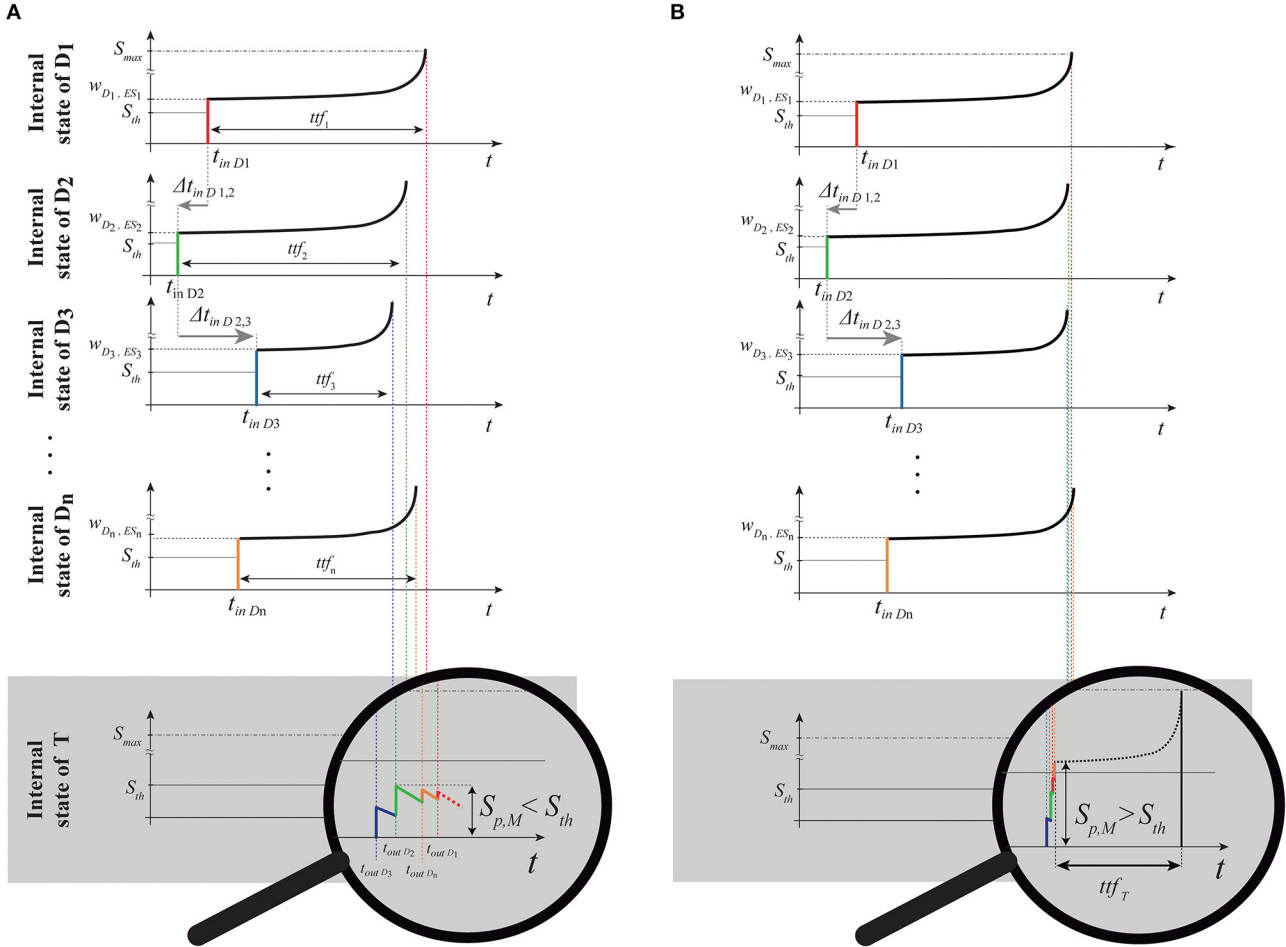
Representation of the same spike train shown in Figures 2, 3 passing through the delay layer and related impact on the internal state of the delay neurons *D*_*i*_ and target neuron *T*. **(A)** Internal neuron representation (*S*_*D*_*i*__) of the absolute spike times (*t*_*inD*_1__, *t*_*inD*_2__,., *t*_*inD*_*n*__) and relative intervals (Δ*t*_*inD*_1,2__, Δ*t*_*inD*_2,3__, ..., Δ*t*_*inD*_*n*−1,*n*__) of the parallel spike train. In this case, the target will not produce a spike because the simultaneity condition at the target is not reached (relative intervals of the input are not coincident with the PPT of the structure); **(B)** we show the response of a structure with a different PPT, to the same parallel input train, which summation tends to the *simultaneity condition*. Consequently, such pattern is detected, and then the spike is generated. It happens after a proper time-to-fire *ttf*_*T*_. To show the summation process to the reader, the diagram of the internal state of the target neuron is zoomed for ease of illustration. Note that the temporal values in this internal representation are symmetrical with respect to Figures 2, 3 since we are representing here the Cartesian temporal axis of times and not a “photography” of the parallel spike train entering the structure.

When the single external input spike goes through the delay layer, the corresponding input weight attenuates/amplifies the pulses accordingly (Figure 4). Hypothesizing the delay neuron *D*_*i*_ is in the resting condition *S*_*D*_*i*__ = *S*_0_ = 0 (where *S*_0_ is the resting potential, see Table 1); after the reception of the spike, the following value will be reached:

(6) $$S_{D_{i}}=A_{ES_{i}}\cdot w_{D_{i},ES_{i}}$$

if the following condition is satisfied for the internal state of *D*_*i*_:

(7) $$\begin{array}{l} S_{D_{i}}\geq S_{th} \end{array}$$

the neuron will produce a spike. It will be done after its time to fire, *ttf*_*D*_*i*__, evoked by the internal state reached by the neuron *D*_*i*_ in according to Equation (2).

Through the latency the LIFL neuron has the extraordinary ability to perform a strength-to-delay transformation, but in the event that all the input weights had the same value and with the assumptions above, the delay neurons would introduce the same lag to each single input spike. In contrast, different weights give rise to different delays: the higher the weight *w*__*D*_*i*_, *ES*_*i*___, the greater the input to the delay neuron (Equation 6) and the lower the delay involved in the spike generation.

Considering an nMNSD of *n* branches, according to its input weight set it presents a *preferential parallel train* (PPT) with respect to the activation of its target, which acts as readout neuron generating a spike in case of recognition. Considering the structure at rest, the arrival of a specific input parallel pattern should produce to its target the same response irrespectively to when it arrives. For this reason, the structure's PPT is defined as a set of preferential relative intervals for each couple of adjacent branches, i.e., the vector of *n*−1 values $\langle \bar{\Delta t_{ES_{1,2}}},\bar{\Delta t_{ES_{2,3}}},...,\bar{\Delta t_{ES_{n-1,n}}}\rangle$, instead of absolute ones. The PPT depends on its input weight set, which reflects how the structure has evolved during the previous learning. Considering all the connections instantaneous (see Table 1) as in the reference work of Susi et al. (2018), the only delays present in the structure are those introduced by the latency feature. Therefore, as a consequence to the introduction of a multi-neuronal pattern to the structure, two different responses are possible:

- The structure is not able to detect the specific input multi-neuronal spike sequence; this is because the delays produced by the input weight set, applied to the current input, do not result in a synchronous target summation;
- The input weight set of the structure generates a set of delays that, in combination with the relative intervals of the current input pattern, verify the *simultaneity condition* at the target, making the target spike, then revealing the detection of the specific input train.

The *simultaneity condition* at the target occurs when the characteristic intervals of the input multi-neuronal spike sequence is coincident (or quasi-coincident) with the PPT of the structure. The more the input characteristic set of intervals fits the structure's PPT, the more the *maximum target summation peak* *S*_*p,M*_ (i.e., the maximum *S* achieved by the target during the summation) will be higher (see Figure 4).

Although the input weight set mediates the mapping of the input pattern in the feature space, the output weight set allows to define the boundaries of the classes. Those 2 weight sets reflect on the positioning and on the shape of the classes in the feature space, respectively (see section 2.6). The PPT consists of a line in the n-dimensional temporal features space, and the output weight set modulates the confidence intervals for a parallel spike train to be recognized with respect to the PPT of the structure. The output weights are chosen so that their sum (i.e., the *target activity level*) is greater or equals to *S*_*th*_:

(8) $$\sum_{i=1}^{n}w_{T,D_{i}}\geq S_{th}$$

i.e., in order to allow the target spike when the favorable “simultaneity condition” is verified. The easier choice for the output weights is to set them to the same value (*w*_*T*,*D*_1__ = *w*_*T*,*D*_2__ =... = *w*_*T*,*D*_*i*__), so that the features have equal weight in the target summation. Alternatively, we can differentiate the degree of importance of each feature in determining a class by giving different output weights to each of the branches (feature relevance).

### 2.3. How the Structure Adapts to New Incoming Patterns

When STDP is active, the structure is ready to learn a new parallel spike train. In this way, one nMNSD is able to identify one class, shaping its boundaries in the feature space according to the examples presented to its input during the training. We define in this section how the structure with STDP activated behaves when a parallel spike train is presented to its input (dynamic behavior).

The adaptive core of the structure resides in the interplay of spike latency and plasticity: the delay *ttf*_*i*_ that characterizes the neuronal pathway *i* is due to the spike latency of the delay neuron *w*_*D*_*i*_, *ES*_*i*__, which in turn is modulated by the neighboring branch(es) through heterosynaptic STDP (an in-depth analysis of such interaction is shown in Susi et al., 2018) when the plasticity is active. In facts, in this case the weight *w*_*D*_*i*_, *ES*_*i*__ is instantaneously influenced in response to a new input parallel spike in the following way:

- Influence of *D*_*i*+1_ on *D*_*i*_
  (9) $$\{\begin{array}{l} \Delta w(D_{i},ES_{i})=A_{+}{\text{e}}^{-\frac{\Delta t\;out_{D_{i+1},D_{i}}}{\tau_{+}}},\text{ for }\Delta t\;out_{D_{i+1},D_{i}}>0\\ \Delta w(D_{i},ES_{i})=0,\text{ for }\Delta t\;out_{D_{i+1},D_{i}}=0\\ \Delta w(D_{i},ES_{i})=A_{-}{\text{e}}^{\frac{\Delta t\;out_{D_{i+1},D_{i}}}{\tau_{-}}},\text{ for }\Delta t\;out_{D_{i+1},D_{i}}<0 \end{array}$$
- Influence of *D*_*i*−1_ on *D*_*i*_
  (10) $$\{\begin{array}{l} \Delta w(D_{i},ES_{i})=A_{+}{\text{e}}^{-\frac{\Delta t\;out_{D_{i-1},D_{i}}}{\tau_{+}}},\text{ for }\Delta t\;out_{D_{i-1},D_{i}}>0\\ \Delta w(D_{i},ES_{i})=0,\text{ for }\Delta t\;out_{D_{i}-1,D_{i}}=0\\ \Delta w(D_{i},ES_{i})=A_{-}{\text{e}}^{\frac{\Delta t\;out_{D_{i-1},D_{i}}}{\tau_{-}}},\text{ for }\Delta t\;out_{D_{i-1},D_{i}}<0 \end{array}$$

As extension of the MNSD, we apply these equations to all delay neurons. Note that, for the first and last branches of the structure, we apply only eq.9 or eq.10, respectively, since they have only one neighbor.

### 2.4. Neuron and Structure Settings

The neuron model used and most of the nMNSD settings presented in this work are based on the reference paper (Susi et al., 2018) and summarized in Table 1.

Importantly, as in Susi et al. (2018), we make two additional assumptions: (1) every time a new parallel input pattern arrives to the structure, all the neurons are at the resting potential *S*_0_, and (2) we consider the STDP constants sufficiently small to avoid interaction among subsequent input sequences. These make possible to analyze the effect of each parallel input to the nMNSD structure separately, both for static and dynamic behaviors.

### 2.5. The Trapezoid Method

The introduction of a new input pattern to the nMNSD structure may make *T* spike. In the affirmative, the spike will be generated after an interval depending on the *ttf*s introduced by delay neurons and target neuron. Then, a *multiple-input single-output* transfer function is associated to each nMNSD.

The *trapezoid method* provides an intuitive representation of the internal mechanism of the structure that allows us to geometrically decompose such transfer function. Looking at the decomposed version of the structure response, we can instantaneously know if a new parallel input train will make the nMNSD target spike, or how we can modify the nMNSD to make it happen. Using a dedicated 2-dimensional nomogram (see Appendix 1 in Supplementary Material), this geometrical method makes possible to evaluate the impact of an input pattern on the *S*_*T*_ of a trained nMNSD of any dimensionality, directly at the input of the structure, without having to execute intermediate steps. This new representation of the detection process provides us with a visual feedback to easily customize the structure's settings to better fit the characteristics of the problem we are facing (e.g., degree of importance of single features in the class definition, compensation of expected feature variance, and so on).

The method envisages as first step to represent all the structure parameters that define its static behavior (as neuron parameters, PPT, and feature relevances), as well as the input arrivals to the target, at the input of the structure through *n* right trapezoids, lying each one on a semi-plane with a real horizontal axis characterized by reversed times (increasing values toward the left, since we are portraying the parallel spike train on the entryway of the structure, see Figure 3). This method allows us to evaluate in a differentiated manner the contribution that each single i-th component of the current input parallel spike train (i.e., *ES*_*i*_) will produce on the *S*_*T*_. The possibility to analyze the nMNSD operation and to optimize the recognition using the simplified visual feedback given by the trapezoids lays the groundwork for the design of stratified nMNSD-based classification systems.

We will present in this section the rationale underlying the trapezoid decomposition, and how it can be obtained. Then, we will show the iter envisaged by the trapezoid method to execute the summation directly at the input of the structure, in two different versions: graphical method and analytical method.

#### 2.5.1. How the Trapezoids Are Drawn?

**Figure 5:**
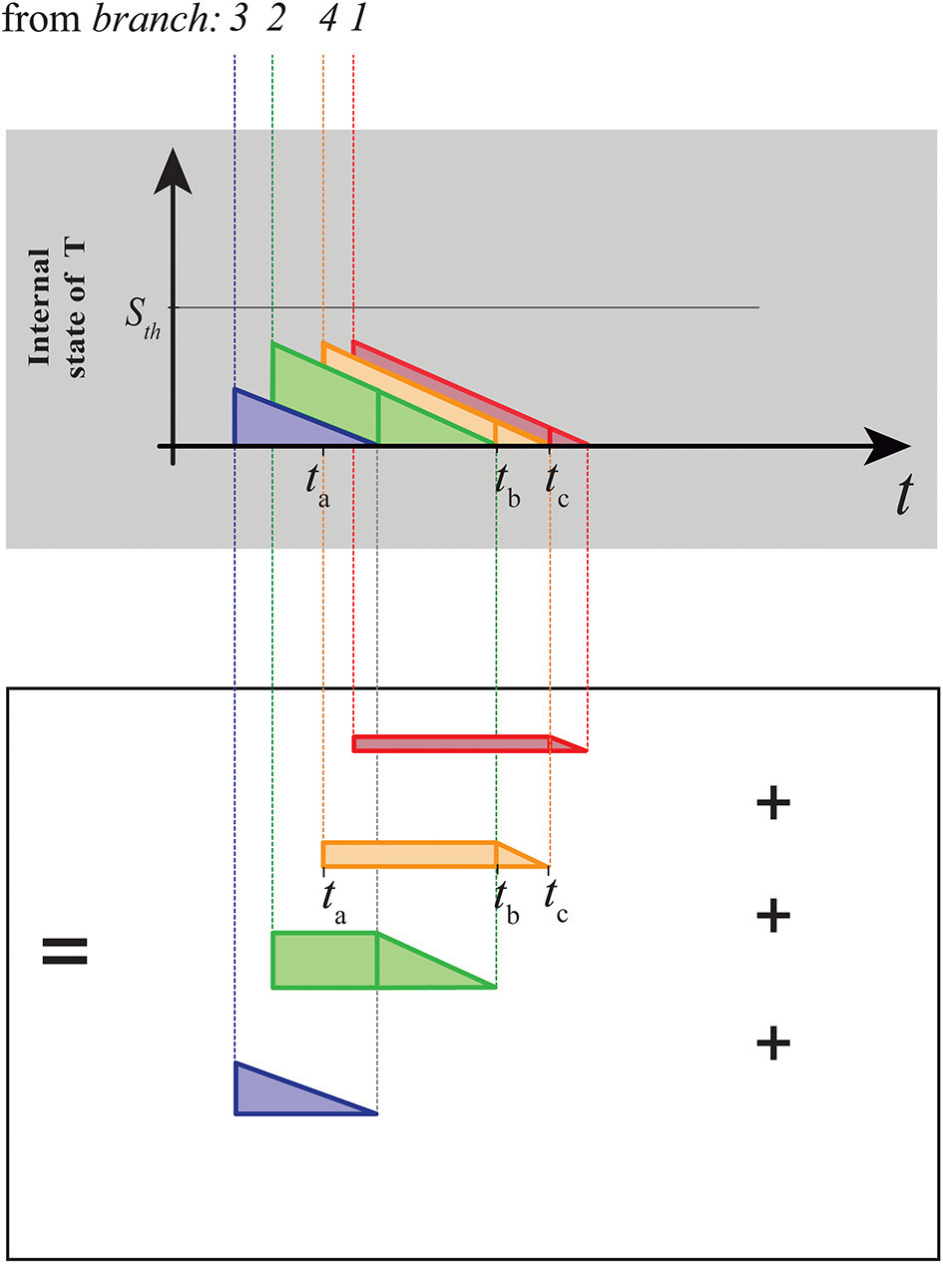
Decomposition of the area subtended by the internal state of the target neuron shown in Figure 4A in terms of trapezoids. Considering the arrival of a generic contribution to the target neuron (e.g., the orange one in A), its advent (at the time *t*_*a*_) will cause an immediate increase of the value of *S*_*T*_ equals to the target weight associated to the afferent contribution. Its contribution on the *S*_*T*_ can be considered an additive constant, until the decay due to the previous contribution is exhausted (interval [*t*_*a*_, *t*_*b*_], i.e., rectangle part). After this, its contribution will start to decrease linearly (with slope *L*_*d*_) until the resting potential is reached (interval [*t*_*b*_, *t*_*c*_], i.e., triangle part).

The trapezoids are geometrical entities that allow us to easily decompose the integration process that takes place into the LIFL in the underthreshold range, as shown in Figure 5. Each trapezoid incorporates information of both the branch of an nMNSD structure (neuronal parameters and input and output weights) and the parallel input that is going through the structure.

Indeed, the generic trapezoid associated to the branch *i* is composed of a rectangle (on the left side) whose base *rect*_*i*_ depends on the current input, adjacent to a right triangle (on the right side) whose base *tri*_*i*_ depends on the structure parameters. The height of the *ith* trapezoid equals to the value of the corresponding *ith* output weight *w*_*T*,*D*_*i*__. The abscissa (time point) of the left limit of the *ith* trapezoid, $\bar{v_{i}}$, is related to the input weights of the delay neurons. Taken as a whole, the $\bar{v_{i}}$ set represents the PPT of the structure.

Like the PPT of the structure, the trapezoids are not constrained to a fixed time point, since they should act at any time, i.e., irrespectively of when the parallel spike train is entering to the network (as noted in section 2.2). Then, without loss of generality, we represent the left extremity of the first trapezoid $\bar{v_{1}}$ as placed to the fictive value *c* and relate the abscissae of the left sides of all the other trapezoids to this value, in the following manner:

(11a) $$\begin{array}{l} \langle \bar{v_{1}},\bar{v_{2}},...,\bar{v_{i}},...,\bar{v_{n}}\rangle = \end{array}$$

(11b) $$\begin{array}{l} =\langle c,c+\bar{\Delta t_{ES_{1,2}}},c+\bar{\Delta t_{ES_{1,3}}},...,c+\bar{\Delta t_{ES_{1,n}}}\rangle = \end{array}$$

(11c) $$\begin{array}{l} =\langle c,c+\bar{\Delta t_{ES_{1,2}}},c+\bar{\Delta t_{ES_{1,2}}}+\bar{\Delta t_{ES_{2,3}}},...,c+\bar{\Delta t_{ES_{1,2}}}+\bar{\Delta t_{ES_{2,3}}}\\ \;+...+\bar{\Delta t_{ES_{n-1,n}}}\rangle \end{array}$$

where:

(12) $$\begin{array}{l} \bar{\Delta t_{ES_{i,j}}}=\frac{1}{w_{D_{j}ES_{j}}-1}-\frac{1}{w_{D_{i}ES_{i}}-1} \end{array}$$

(see Appendix 2 in Supplementary Material). Note that using Equation (11a) and (11c), we can relate the abscissae of the trapezoids to the PPT of the structure, in terms of preferential relative intervals $\langle \bar{\Delta t_{ES_{1,2}}},\bar{\Delta t_{ES_{2,3}}},...,\bar{\Delta t_{ES_{n-1,n}}}\rangle$. In this way, we are operating a geometrical transformation to represent the *S*_*T*_ diagram to the input of the nMNSD, taking in account the effect the *ES*s will undergo once they cross the structure.

We introduce the concept of *crossing order* 〈*ref*_1_, *ref*_2_, ..., *ref*_*k*_, ..., *ref*_*n*_〉, i.e., the set of integer numbers which indicates the sequence of the branch indices which spikes will progressively arrive to the target neuron (to give an example, 〈3, 2, 4, 1〉 in Figure 5). To decompose the target summation in trapezoids, the first geometrical object to be drawn is the one associated to the branch *ref*_1_, i.e., the branch which contribution will arrive for first to the target. It will lack of the rectangle part, consisting then on a simple right triangle. It has a slope equals to the underthreshold decay of *T* (i.e., *L*_*d*_), as all the other triangles of the chart, so that:

(13) $$\begin{array}{l} tri_{ref_{k}}=\frac{w_{T,D_{ref_{k}}}}{L_{d}} \end{array}$$

To draw the set of rectangles, we have to take in account the arrival order of the contributions to the target. Considering Figure 5, we note that the length of the rectangle associated to a generic branch is given by the interval between the arrival of its own contribution to the target and the end of the previously arrived trapezoid/triangle (i.e., *t*_*a*_ and *t*_*b*_ respectively, if we consider the orange trapezoid in Figure 5). Considering the correlate of such temporal distance at the input of the structure (transformed by the eq.12), we obtain the set of rectangle lengths by iterating the following formula, for *k* = 2, 3, ..., *n*:

(14) $$\begin{array}{l} rect_{ref_{k}}=rect_{ref_{k-1}}+tri_{ref_{k-1}}-\left(\bar{v_{ref_{k-1}}}-\bar{v_{ref_{k}}}+t_{ES_{ref_{k}}}-t_{ES_{ref_{k-1}}}\right) \end{array}$$

where, obviously, *rect*_*ref*_*k*−1__ = 0 when we compute *rect*_*ref*_2__. If the interval between two arrivals is long enough to allow *S*_*T*_ to fully discharge, then the related trapezoid will lack of the rectangle part, as the first trapezoid. To obtain the complete set of trapezoids, we have just to complement the trapezoids, adding the triangles defined above at the right end of the rectangles generated.

#### 2.5.2. Iter Description

**Figure 6:**
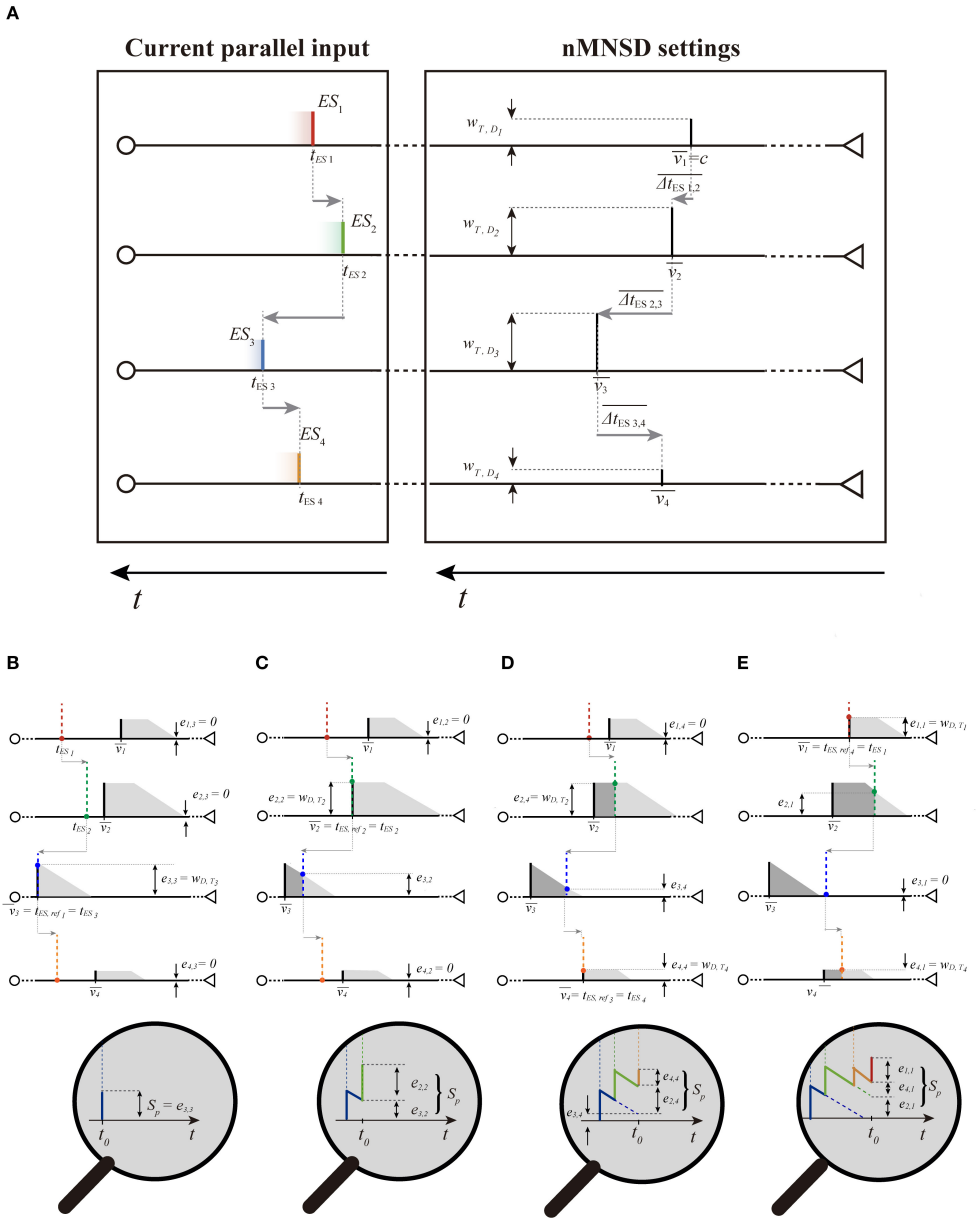
Target summation executed with the trapezoid method. **(A)** The PPT of a parallel spike train of order 4 (left) moving toward the preferential parallel train (PPT) of the nMNSD settings; the two blocks are here represented separately for ease of understanding. Note that the time axis has to be considered reversed in both the blocks (as in Figure 3), since we are portraying the parallel spike train in motion. Envisaging the parallel train sliding toward right we can notice that ESs will cross the PPT of the structure with the following ES order (i.e., the following crossing order): 〈3, 2, 4, 1〉. In **(B–E)**, we give a “stop-motion” representation of the target summation executed with the trapezoid method, showing both the parallel spike train and the trapezoid set of the structure on the same chart (with related zoomed representation of *S*_*T*_ below). Except for the trapezoid associated to the first crossing step (which always lacks the rectangle part, as explained in the text), a generic trapezoid has the rectangle length determined by the time difference between its arrival and the end of the trapezoid associated to the last contribution arrived to the target; once the rectangle parts have been drawn, the triangle parts can consequently traced to the right of the rectangle parts to obtain the complete trapezoids. Using the trapezoid method, the efficacies *e*_*i, re*_*f*__*k*__ to the state of the target neuron *S*_*T*_ are directly noticeable on the input of the structure, since they are represented by the heights of the intersections between the ES and the upper perimeter of the related trapezoid (colored dots). At this point, the summation can be easily decomposed in the contribution of each branch. In the zoom below, the reader can ascertain for the first three crossing steps (*ref*_1_ = 3, *ref*_2_ = 2, *ref*_3_ = 4) that the computation of the *S*_*p*_ through the trapezoid method is equivalent to the one obtained by the classical target summation at the target neuron.

Now that we know how to draw the trapezoids on our chart, we can go back to analyze the passage of the train in the structure looking at the input of the structure only. We conceive the *n* trapezoids placed on the abscissae $\langle \bar{v_{1}},\bar{v_{2}},...,\bar{v_{i}},...,\bar{v_{n}}\rangle$, and the current input parallel spike train, characterized by the absolute times 〈*t*_*ES*_1__, *t*_*ES*_2__, ..., *t*_*ES*_*n*__〉. For ease of representation, we consider the spike train in motion toward the structure (from left to right, see Figure 6A, left), locked on their Cartesian references, as if they too were moving toward the nMNSD.

Following this method, we developed an interactive visualization and optimization system of nMNSD structures, available at www.github.com/LCCN/Frontiers2021.

##### 2.5.2.1. Graphical Method

From the parameters of the nMNSD, we are able to draw the PPT of the structure on the trapezoid chart; once we know the input parallel input pattern, we can complement the graph with the related trapezoid set as described above. The method envisages as preliminary step the detection of the *crossing order*. We can alternatively visualize it as the sequence of the branch indices which input spikes will progressively cross the left side of their related trapezoid, during the entrance toward the structure. The crossing event related to *ES*_*i*_ allows us to represent the spike contribution on the target neuron from the branch *i*. Note that the crossing order does not reflect the indices of the branches, which *ESs* ordinately arrive to the related delay neuron, but the sequence of the target arrivals evoked by the *ESs* (the one thing does not imply the other). The crossing order can be graphically individuated by rigidly translating the parallel input train toward the trapezoids, and reporting the sequence of crossing (see Figures 6B–E). Each *crossing step* of the *ES*_*i*_ on the associated trapezoid (left extremity) represents the arrival of the contribution from the branch of order *i* to the target; each crossing step corresponds to a relative maximum on the membrane potential of the target *S*_*T*_ (i.e., a summation peak *S*_*p*_). Since *S*_*T*_ depends in general also on the past arrivals, taking in account a generic crossing step *ref*_*k*_, we can represent the related *S*_*p,ref*_*k*__ as the sum of the residual contributions given by each target arrival. Such contributions are called *target efficacies* (indicated as *e*_*ES*_*i,ref*_*k*___) and are represented by the heights of the intersections of the prolongations of each *ES* of the train with the related trapezoid. Note that a set of target efficacies 〈*e*_*ES*_1,*ref*_*k*___, *e*_*ES*_2,*ref*_*k*___, ..., *e*_*ES*_*n,ref*_*n*___〉 (and consequently a *S*_*p*_) can be calculated for each one of the *n* crossing steps. Obviously, since the value *ref*_*k*_ is the branch number which related spike arrives *k*−*th* to *T*, when we consider the crossing step associated to *ES*_*i*_, its target efficacy is represented by the full *w*_*T*,*D*_*i*__ (i.e., *e*_*ES*_*i,ref*_*k*___ = *w*_*T*,*D*_*ref*_*k*___ when *ref*_*k*_ = *i*). That said, for each crossing step, *S*_*p,ref*_*k*__ can be calculated by summing up the *e*_*i,ref*_*k*__ related to all the parallel spike train components. For a complete parallel spike train, we call *S*_*p,M*_ the maximum among all the *S*_*p*_s. If in almost one of these steps, the value *S*_*p,ref*_*k*__ is greater than *S*_*th*_, then the target neuron will produce a spike, signaling the detection of the input pattern by the nMNSD structure. The process is illustrated graphically in Figure 6, and analytically in Supplementary Table 1 of the Appendix 2.

#### 2.5.3. Will the Target Produce a Spike? And When? Beyond the Behavior of an Isolated nMNSD

As already shown in section 2.2, a spike will be produced by the target neuron only if, at least for one of the crossing events, results that *S*_*p,ref*_*k*__>*S*_*th*_, signaling the recognition of the pattern. Analytically speaking, each new incoming pattern will be able to evoke an output spike on the target only if the following condition is satisfied:

(15) $$\begin{array}{l} S_{p,M}=max\left(S_{p,ref_{1}},S_{p,ref_{2}},...,S_{p,ref_{k}},...,S_{p,ref_{n}}\right)\geq S_{th} \end{array}$$

**Figure 7:**
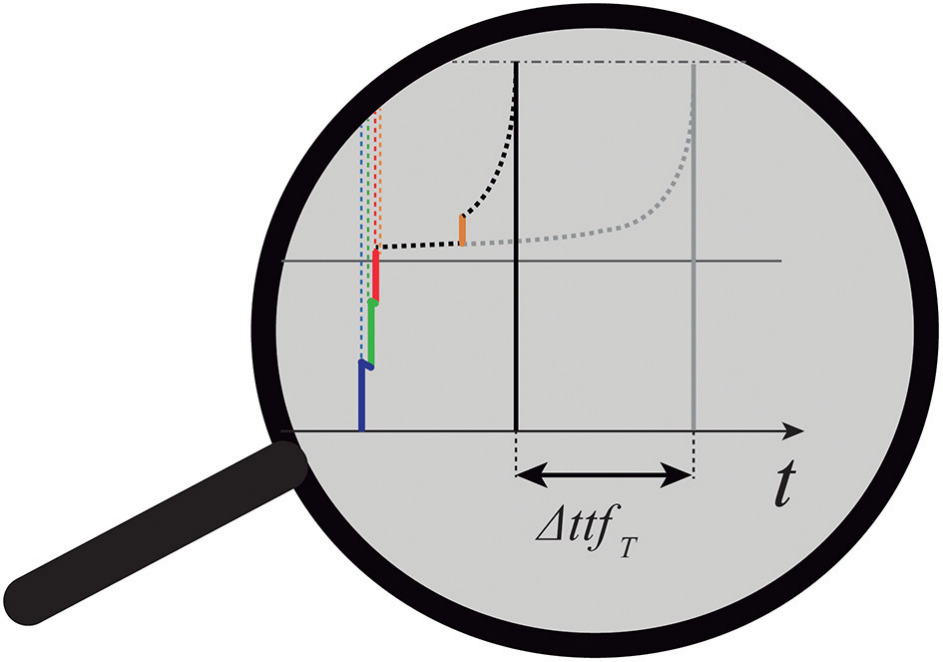
Peculiar case of target summation in which one *ES* arrives to *T* while it is already in the active mode.

The spike will be generated after a proper time-to-fire *ttf*_*T*_ (Equation 2). To calculate this value, we have to discern among two cases:

- Case 1: No *ES* of the parallel spike train will arrive to *T* during it is in the active mode. In this case, the *ttf*_*T*_ can be easily calculated using Equation (2), considering *S*_*p,M*_ as *S*.
- Case 2: Some *ES* of the parallel spike train will arrive to *T* during it is already in the active mode (see Figure 7). Such case necessitates further attention; see (Susi et al., 2018, Supplementary Material, Par.1).

In order to avoid overcomplicating the analysis, we study here the first case, assured by imposing the following condition on the output weights:

(16) $$\begin{array}{l} \left(\overset{n}{\underset{i=1}{\sum }}w_{T,D_{i}}\right)-w_{T,D_{j}}<S_{th}\text{, for each}j\text{ chosen between }\left[1,n\right] \end{array}$$

On the other hand, their sum should be at least equal to *S*_*th*_, as previously stated (see Equation 8). The time of spike generation gives us a score of the goodness of the input, and it can be used as further information to build more complex configurations of nMNSD, as discussed in section 2.7.

### 2.6. Shape of the Hypervolume

Among the many architectural parameters of the nMNSD, we can differentiate between those involved in the recognition of a pattern and those involved in its subsequent signaling. As indicated in section 2.3, the input weights represent the PPT of the nMNSD, then they have to do with the actual recognition of the input pattern. In the n-dimensional feature space, the PPT of the nMNSD is represented by the line ζ, with slope of 45° with respect of each of the axes and passing through the point determined by the following coordinates:

(17) $$\begin{array}{l} \frac{1}{w_{D_{1},ES_{1}}-1},\frac{1}{w_{D_{2},ES_{2}}-1},\frac{1}{w_{D_{3},ES_{3}}-1},...,\frac{1}{w_{D_{n},ES_{n}}-1} \end{array}$$

(for an in-depth explanation, see Susi et al. (2018)). To understand the signaling phase of a pattern, let us consider a volume in the n-dimensional feature space (i.e., a hypervolume), consisting of an augmentation of the PPT such that if the set of arrival times of a pattern falls into it, the MNSD produces a spike. Such augmentation represents the tolerance of the structure, i.e., the error margin the nMNSD admits from the PPT of the parallel input to consider it recognized. To modify the hypervolume, we can act on the target weights and *L*_*d*_ (of the target neuron):

- Target weights allow to introduce a selective tolerance with respect to a single feature. This is useful when we have fluctuations of the values of a determined involved feature;
- *L*_*d*_ modulates the tolerance of the structure with respect to all its features: the higher (lower) the *L*_*d*_, the more selective (robust) the structure becomes to the jitter. If we have equals *w*_*T*,*D*_1__, we have a hypercylinder as hypervolume, whose radius depends on *L*_*d*_.

### 2.7. Toward Complex nMNSD-Based Classification Systems: Multiclass Classification and Layering

The nMNSDs can be used for multiclass classification by assigning an nMNSD to each class of the problem. The training can be made separately for each nMNSD structure, paying attention to sufficiently differentiate the domains of the classes, to avoid that more than a target will fire for the same input, causing indeterminacy of the belonging of the pattern to one of the two classes. Since each structure is represented by its own trapezoid set, the refinement of the hypervolumes associated to the classes can be achieved by minimizing the intersection area between the different trapezoid sets. In cases where the intersection of classes is unavoidable, the evaluation of the *ttf*_*T*_ can be informative since an anticipated spike is often representative of a better fit with the class represented by the structure that produced it.

**Figure 8:**
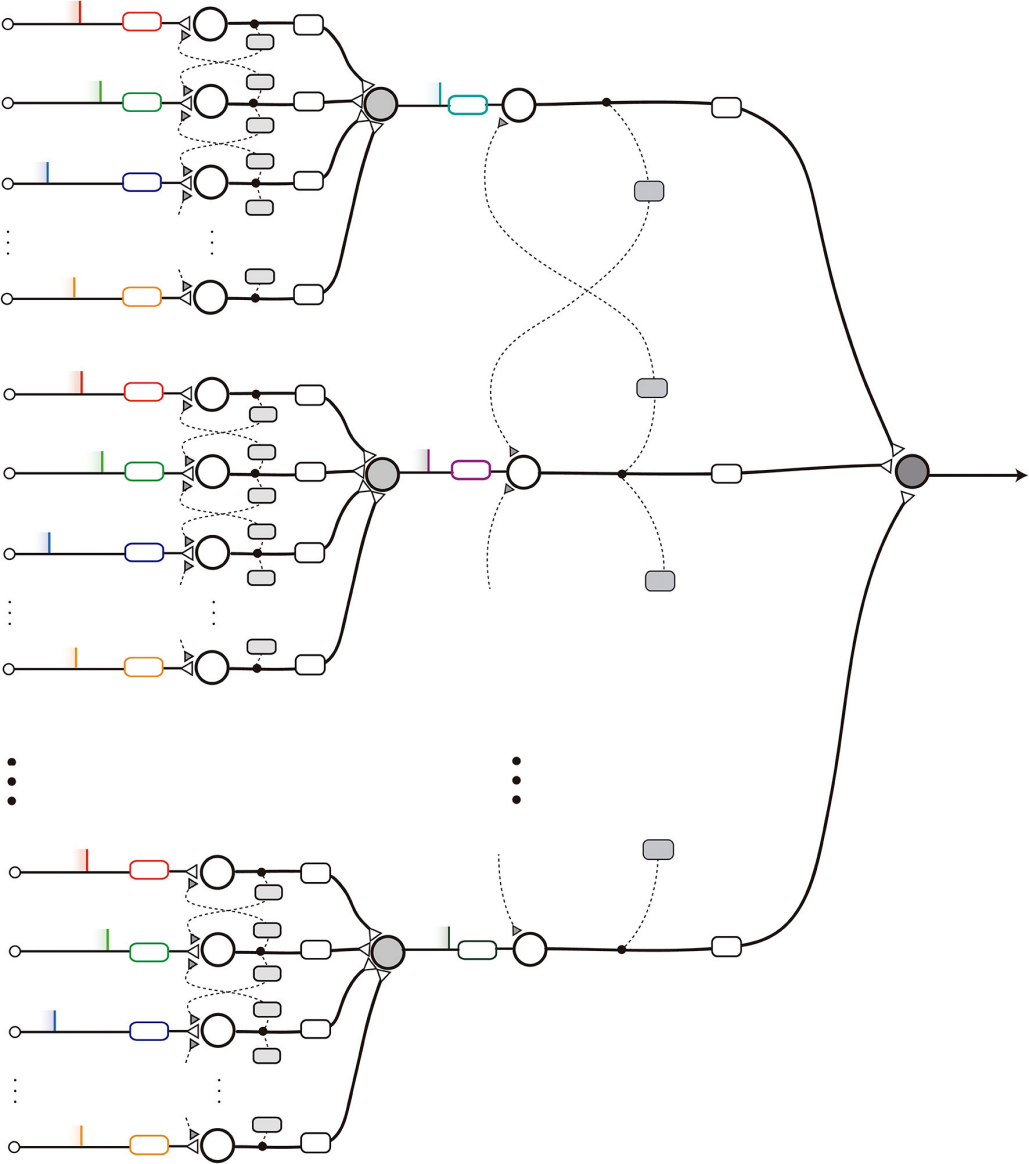
Scheme of a layered nMNSD-based system.

Another configuration can be obtained by parallelizing the nMNSD structures to analyze their output together. Each nMNSD of the same layer will produce a spike on a certain instant, which together will form a new parallel spike sequence. nMNSDs of subsequent layers will be gradually trained on the parallel spike sequences evoked by previous levels. Note that in each layer one should adapt the parallel patterns to fall within the proper range of action (see Figure 8).

## 3. Results

In order to show the improved usability of the nMNSD tool to tackle pattern recognition problems with respect to the MNSD, we test the structure on two specific datasets. First, we face again the classification problem presented in Susi et al. (2018) (section 3.2) concerning the recognition of No-Go patterns from a patient executing the Go/No-Go paradigm, and finally we benchmark the nMNSD on static inputs using the MNIST dataset of handwritten digits to find pros and cons compared to other SNN-based classification methods. The simulations are performed using an event-driven implementation of the nMNSD structures in Matlab2019b environment.

### 3.1. Go/No-Go Paradigm

The data of this first test concern a Go/No-Go paradigm characterized by a 70/30 presentation ratio. The study is ongoing and conducted at the Laboratory of Cognitive and Computational Neuroscience of Madrid. It involves 67 healthy and right-handed subjects (age range 13–17 years old), without previous history of psychiatric or neurological conditions, neither psychopharmacological treatment nor drug intake. High-density magnetoencephalography (MEG) signals were obtained from 306 channels (102 pairs of planar gradiometers and 102 magnetometers) with an Elekta Neuromag Vectorview system situated in a magnetically and electrically shielded room. Only the 102 Magnetometers were used to carry out the analysis.

**Figure 9:**
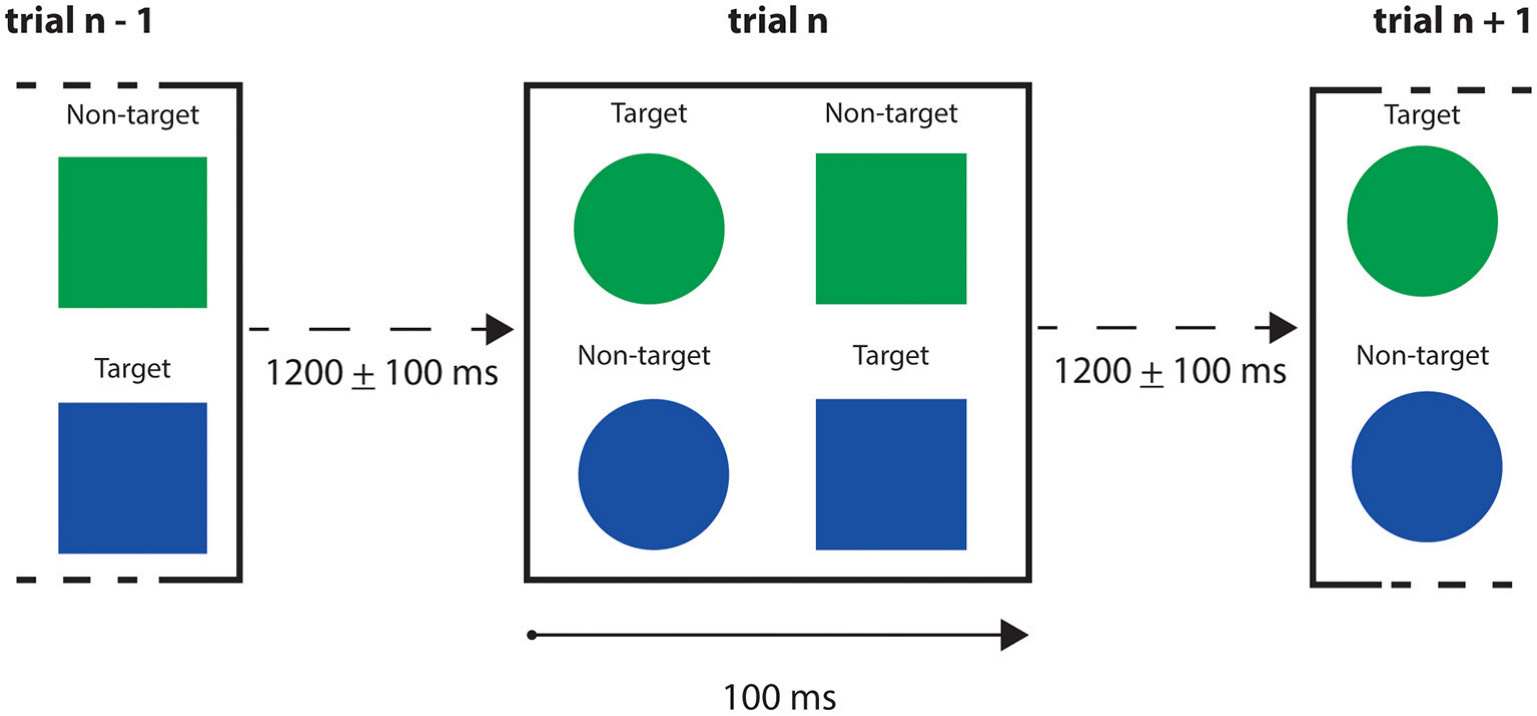
Go/No-Go trials schematic representation. Trial's exposition time for the stimuli was 100 ms, with a *stimulus onset asynchrony* (i.e., the time interval between two trials) between 1,100 and 1,300 ms. The stimuli presented were blue and green circles and squares. Participant was instructed to press a button when a blue square or a green circle appeared, and not to press for green squares and blue circles.

The MEG data from the subject with the highest performance in both Go and No-Go conditions have been chosen for further analysis (we selected the best performer to minimize the risk of having unintentional or random responses in our dataset). We finally considered for each trial the time interval < 400*ms* after the stimulus presentation to exclude the premotor response (Deecke et al., 1976; Ikeda et al., 2000). A schematic representation of the task is given in Figure 9, and a resume of the subject performance in Table 2.

**Table 2:** Go/No-Go task.

**Total**	**Correct**	**Accuracy**	**Accuracy**	**Averaged**
**inhibitions**	**inhibitions**	**inhibitions**	**response**	**reaction time**
103	101	98.05%	100%	546.43 ms

*Resume of the performance of the selected participant*.

**Figure 10:**
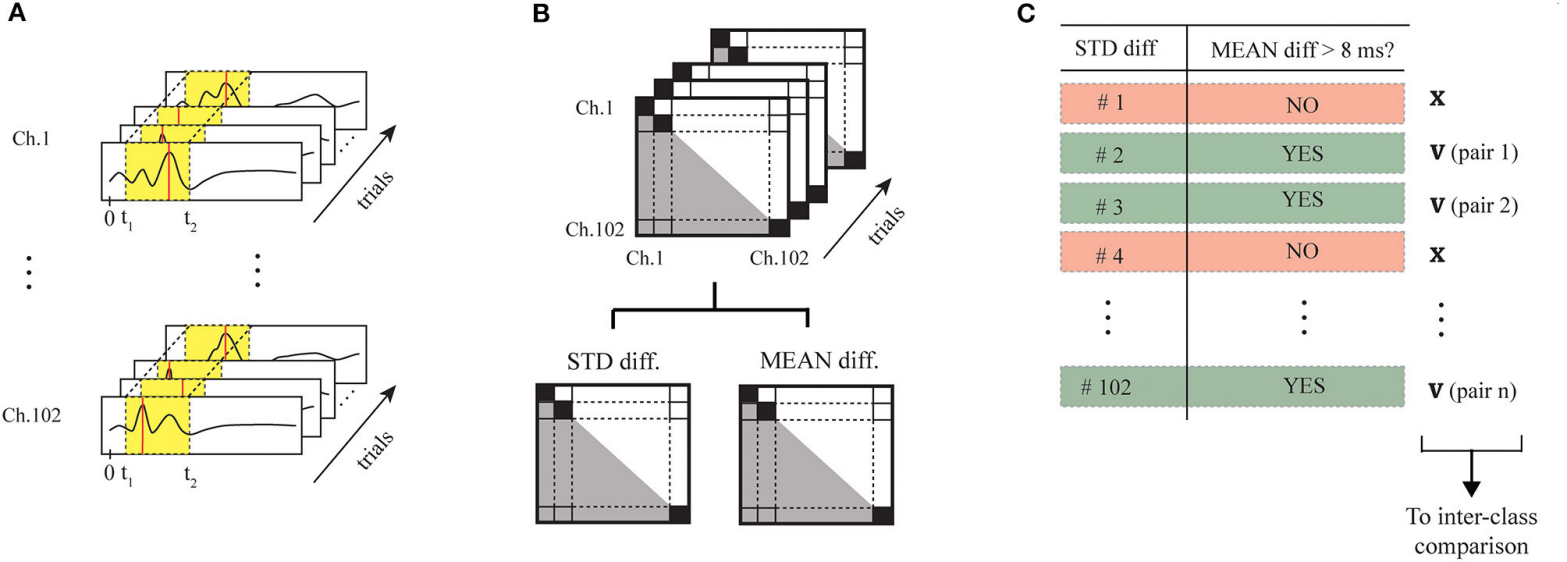
Processing pipeline for the selection of the eight informative magnetometers. Considering the time series during the window of interest (in yellow), we executed the following steps first for the No-Go trials and then for the Go trials: **(A)** detected the maximum peak on each MEG channel, for each of the trials; **(B)** we computed the time difference considering each combination of magnetometers, and extracted mean and standard deviation of the time difference on each pair of MEG sensors along the trials; **(C)** we selected the couples of sensors starting from those characterized by the lowest standard deviations and a mean ≥ 10*ms*. Inter-class discriminative power has been then guaranteed by comparing the 2 sets of sensor couples selected for the 2 classes, and removing possible pairs of sensors duplicated in the 2 classes if they presented the same mean.

We preliminarily selected for each of the two classes (No-Go and Go trials) the networks of informative magnetometers, evaluating the patterns of occurrence of absolute maxima in the reference time window. We evaluated their discriminative power by computing their intra-class stability and inter-class independence. In Figure 10, the processing pipeline performed for each of the classes is illustrated. On the bases of previous studies (Amirali et al., 2018), we quantified stability and discriminative power of the magnetometers using different versions of the time series (broadband unfiltered time-series, *alpha*-filtered time series, and *theta*-filtered time series), obtaining better results with the original broadband time series. To differentiate the two classes, we finally chosen the eight magnetometers indicated with the IDs listed in Table 3.

**Table 3:** List of the IDs of the selected MEG magnetometers representative of the two classes: No-Go and Go.

**Magnetometer ID:**	0231	0241	0911	0921	1521	1541	1631	1911

The nMNSD has been trained (75 trials for each class) and then tested (20 trials for each class) to recognize No-Go patterns using the instants corresponding to the maximum amplitudes in the time window of the premotor response. We repeated a whole training cycle (learning + validation) varying the parameters *L*_*d*_ [0.005, 0.08], *A*_+/−_ [0.001, 0.04] and τ_+/−_ [5, 8] to find the optimal nMNSD settings (which we call *best learners*). Using an *Intel(R) Core(TM) i5-8250U CPU, 8 GB RAM*, each complete training cycle lasted an average of 10 ms, and the recognition of a single No-Go pattern lasted only 0.1 ms. The simulations individuated a rich area with a satisfactory trade-off between average Accuracy, Precision, and Recall (up to 0.75, 0.76, and 0.72, respectively).

**Figure 11:**
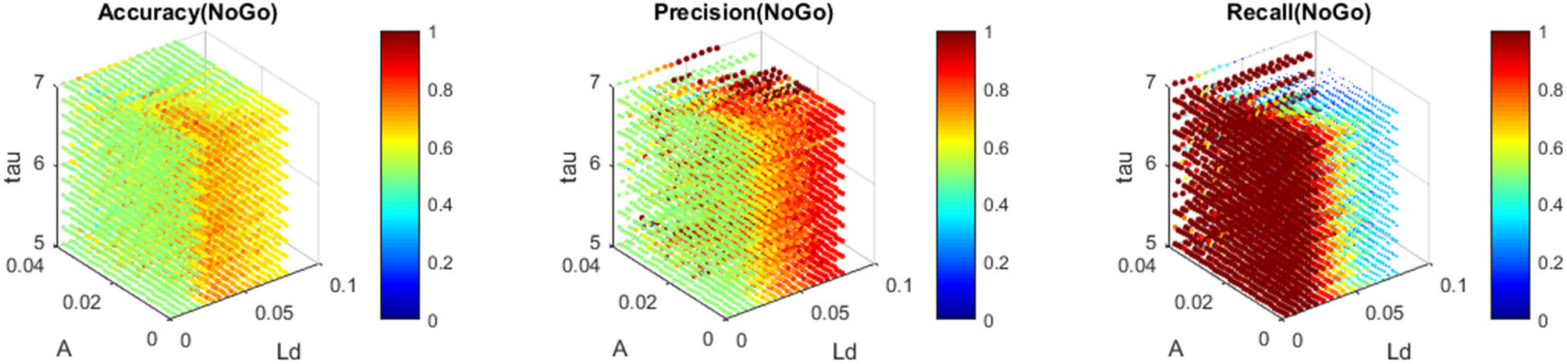
Accuracy, Precision, and Recall obtained with the trained nMNSD (No-Go structure) around the optimal parameters. Each of the colored point represents the performance of the nMNSD trained with the parameters indicated in the axes.

**Figure 12:**
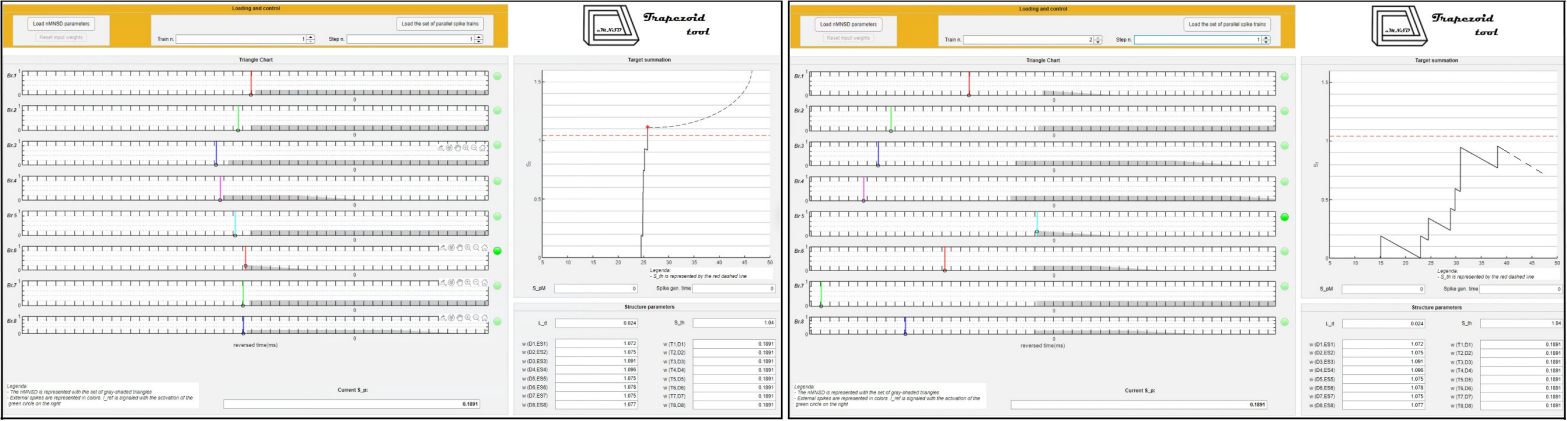
Representation on the trapezoid chart of the No-Go structure after the training, when crossed by 2 parallel spike trains. **Left**: The No-Go structure is stimulated with a No-Go trial (correctly recognized). **Right**: The No-Go structure is stimulated with a Go trial (that is not recognized by the structure).

In Figure 11, we represent the classification performance obtained with the different settings of the trained nMNSD, and in Figure 12 the representation on the trapezoid chart of the trained No-Go structure, when crossed with a No-Go trial and then with a Go trial.

Beside the fact that this represents an improvement with respect to the recognition performances achieved in our previous work, we presented a new methodology, able to take into account a greater number of features, and the representation of the trained structure in the trapezoid chart. Importantly, since we analyzed the time series during the premotor response (<400*ms* while average response of the subject happened at 546.4 ms), and the delay of our system is negligible, our method allows to perform the classification before the action is made from the subject. This opens up interesting scenarios for the action control in critical decisions.

In addition to the visualization of the nMNSD settings with respect to a given parallel spike train, the realized toolbox leaves room for the improvement of the tuning of the structure through the positions of the *trapezoids* and allows the user to analyze the *feature relevances* to differentiate the impacts evoked by the different features of the problem. Obviously, this method can be extended to other types of problems, even in areas other than neuroscience.

### 3.2. MNIST Database

In this second test, we adapted the nMNSD structure to classify 2D static inputs using the grayscale images of MNIST database (LeCunn et al., 1998). The original images are composed of 28 × 28 pixels and each pixel spans the range [0–255]. We created two additional subsampled versions of the images by grouping neighboring pixels in *fields*. Images are encoded in the input layer through the time-to-first-spike of the assigned neurons, using the following intensity-to-latency relation:

(18) $$\begin{array}{l} t_{i}=\frac{I_{max}-I_{i}}{I_{max}}\cdot 25 \end{array}$$

where *I*_*max*_ is the maximum value of intensity of a pixel/field, *I*_*i*_ the actual value of intensity of the pixel/field, and 25 ms of simulated time is the maximum latency encoded (corresponding to an “unwritten” pixel/field ). This does not need any of the preprocessing steps commonly used in SNNs (e.g., Gabor filters). We repeated a whole training cycle (learning + validation) varying the parameters *L*_*d*_, *A*_+/−_ and τ_+/−_ in the same ranges cited in section 3.1 to find the optimal nMNSD settings (i.e., the parameters of the *best learners*).

**Figure 13:**
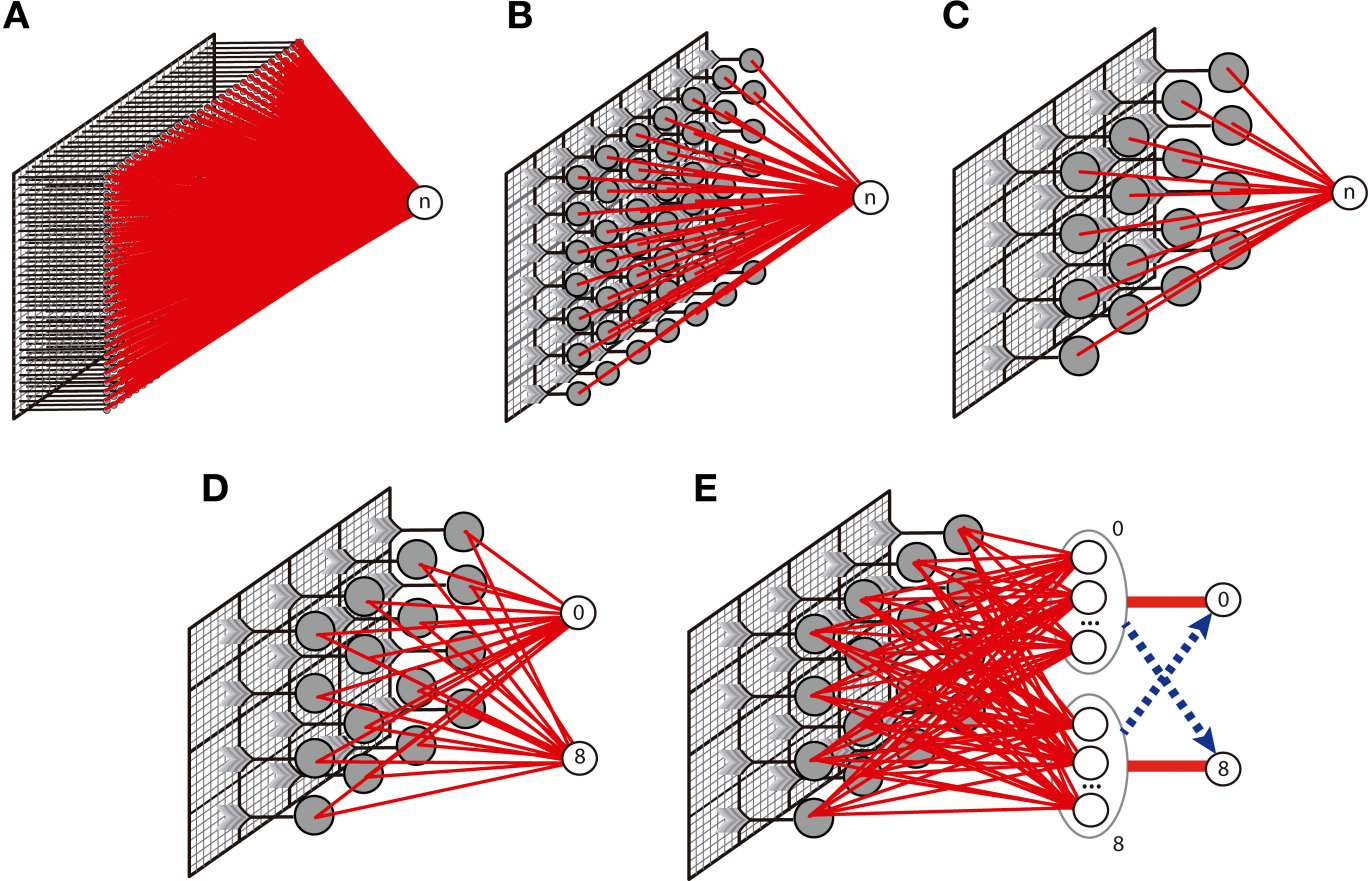
Description of the different network topologies tested. In **(A)**, the nMNSD is fed with the original image (28 × 28 pixels); in **(B)**, the pixels are grouped in 49 (7 × 7) fields; in **(C)**, the pixels are grouped in 16 (4 × 4) fields. one-versus-one (OvO): **(D)** Original configuration and **(E)** enhanced version (with inhibitory bundles in blue).

We implemented three versions of the nMNSD network (i.e., A, B, and C), illustrated in Figure 13. While in configuration A we simply attributed each pixel of the original image to one different input neuron of the nMNSD, in configurations B and C we grouped the branches in adjacent fields composed of 4 × 4 and 7 × 7 pixel each, respectively, and assigned to them one different input neuron of the nMNSD, using the mean value of intensity of the field and coded this number to the input neurons using Equation (18). Heterosynaptic lateral connections are activated between adjacent neurons during learning and disabled during test.

We tested the nMNSD considering the two strategies *one-versus-all* (OvA) and *one-versus-one* (OvO).

For the OvA, the recognition system is composed of one nMNSD structure, which is trained to detect a digit vs. the complementary ones. We considered the digit “1” for simplicity, since it is the one with the greater number of examples: *n*_*LEARNING*_=6742, *n*_*TEST*_=1134. For each combination of the parameters *L*_*d*_, *A*_+/−_, and τ_+/−_ explored, we executed both training and test with balanced quantities of examples for preferred and non-preferred classes (for learning: 6742 images for digit “1” and as many images among the digits [0, 2−9], considering the first 60000 trials of the MNIST dataset; for test: 1134 images for digit “1,” and as many among the remaining trials for the other digits, considering the last 10000 trials of the dataset). Once chosen the structure parameters and the input weights (best learners), we optimized the output weights using the *fminsearch* algorithm (based on the *Nelder–Mead* search method Nelder and Mead, 1965; Lagarias et al., 1998), obtaining an average improvement of 5% in accuracy, arriving to values of 79, 82, and 93% for the A, B, C topologies, respectively. The results are shown in Table 4.

**Table 4:** Comparison of one-versus-all (OvA) performance obtained with the three structures implemented.

**Configuration**	**Learning time** **(for training cycle)**	**Accuracy**
A	300*s*	0.79
B	1.35*s*	0.82
C	0.4*s*	0.93

For the OvO, the recognition system is here composed of two parallel 4 × 4 nMNSD structures of type C, each one trained to recognize one digit. We executed the training considering 5421 images per class. Then we optimized the output weights, and finally tested the network using 837 images per class. To resolve the cases in which more than 1 target produces a spike during the recognition, we evaluated two different strategies: (1) we considered recognizing the digit associated to the target which produces a spike for first; (2) we used 5 redundancy nMNSDs for each class and provided each target with a bundle of “muting” connections that aim to inhibit the complementary final target during its latency period. The topology for this latter variant, which we call *OvO enhanced*, is described in Figure 13E. In this second case, the learning has been carried out initializing input weights and internal states to random values to broaden the differentiation between the redundancy modules. Both these strategies increased the system performances; while the first strategy results faster to train, the second needs an additional supervised step (for the inhibitory neurons), but leads to better results. The latter structure achieved an accuracy of 95%.

The learning times for each training cycle (using the same computer specified in section 3.1) showed significant variations with respect to the topology used. The results are shown in Table 5. Differently, the classification time for each presented pattern do not varies substantially with respect to the topology used and is equal to 27 ± 8μ*s*.

**Table 5:** Comparison of one-versus-one (OvO) performances obtained with the two methodologies implemented (best accuracies).

**Configuration**	**Learning + optimization times** **(for training cycle)**	**Accuracy**
D	[0.4 + 5]*s*	0.91
E (OvO enhanced)	[0.45 + 6.5]*s*	0.95

Regarding the OvA configuration, the grouping operation leads to a drastic reduction of the learning times and to an increase of accuracy. Regarding the OvO configuration, the enhanced version reported the best results. The values of accuracy are notable considering the very simple network configuration (17 neurons and 32 connections for the OvA*-C*, 28 neurons and 180 connections for the OvO*-E*).

Compared to existing character recognition systems based on SNN, the nMNSD showed a state of the art level accuracy. At the same time, the system is composed of a very limited number of neurons and synapses if compared to today's SNN-based recognition systems (see Kheradpisheh and Masquelier, 2020) for a useful report) and the recognition process consists of only one spike per neuron. Taken together, these characteristics testify the proneness of the nMNSD to time- and energy-efficiency, two of the most desirable features in electronic systems (see Göltz et al., 2020).

## 4. Discussions and Conclusions

We presented the nMNSD structure, which is a generalization of the MNSD to any number of inputs. After the explication of the structure operation, we discussed some aspects to improve the practical usability of the nMNSD as classifier tool. In addition, we have illustrated the *trapezoid method* that is a reduced method to analyze the recognition mechanism operated by the nMNSD in response to a specific input sequence. This method is useful to represent the internal processing mechanism of a specific nMNSD, especially when the number of branches is greater than three, i.e., when the feature space becomes not trivially representable. A visualization toolbox based on this method is available at www.github.com/LCCN/Frontiers2021.

The first application showed in this paper regards the classification of brain signals. We applied the nMNSD to a problem previously faced with the classical MNSD from the same authors, showing the improvement in classification performances achieved with the nMNSD structure with respect to the simple MNSD, due to the possibility of encoding a greater number of features, and the support in the “neural design” brought by the visualization tool realized. Finally, we tested the performance of the nMNSD on the MNIST dataset. Regarding this application, we developed different versions of the nMNSD, including one based on a redundancy layer, which showed the best performance in terms of accuracy, at the cost of a greater number of spikes in the network, which would results in greater power dissipation in hardware implementations. Nevertheless, considering that the encoding strategy of the nMNSD is based on single spikes and not on spike-rate, the dissipated power would still be low and the impact is minimal, so the benefits of this change far outweigh the negatives.

Among the most important highlights on the presented method, there are:

- The possible use of the information given by the latency of the target neuron in case of indetermination in a multiclass scenario,
- The possibility to encode the impact of each feature in the target summation for the determination of a class (i.e., *feature relevance* property),
- The configuration of different nMNSDs to be used in a multiclass problem.

Recent trend in sensor technologies are pointed to resource-efficient algorithms for high-speed learning and online recognition, to be implemented directly on the sensors (Kasetty et al., 2008; Iacoviello et al., 2015; Cardarilli et al., 2020). Our results show that the nMNSD proficuously taken in consideration for pattern recognition applications requires low power consumption and low training times.The simplicity and low computational cost of this methodology suggest a large-scale implementation for real-time learning and recognition applications in several areas.

## Data Availability Statement

The datasets generated for this study are available on request from the corresponding author.

## Ethics Statement

The studies involving human participants were reviewed and approved by Complutense University of Madrid. Written informed consent to participate in this study was provided by the participants' legal guardian/next of kin.

## Author Contributions

GS and CM designed the computational framework. GS, FM, and EP conceptualized the paper. LA-T and FM provided and analyzed brain data. GS, LA-T, FM, EP, and CM wrote the paper. All authors contributed to the article and approved the submitted version.

## Conflict of Interest

The authors declare that the research was conducted in the absence of any commercial or financial relationships that could be construed as a potential conflict of interest. The reviewer GC declared a past co-authorship with one of the authors GS to the handling editor.
