# Supplementary material for: nMNSD—A Spiking Neuron-Based Classifier That Combines Weight-Adjustment and Delay-Shift
Source: Front Neurosci. 2021 Feb 19;15:582608. doi: 10.3389/fnins.2021.582608 (PMC7933525; doi:10.3389/fnins.2021.582608)
Supplement: Supplementary file 1 [file Data_Sheet_1.PDF]

Supplementary Material

Appendix 1. The trapezoid tool

The trapezoid method has been implemented as ”Matlab App” under the Matlab environment (Mathworks). In Fig. S1 the main menu of the tool is illustrated. The nomogram lies on a semi-plane with a real and horizontal axis characterized by reversed times (increasing values toward the left, since we are portraying parallel spike train on the entryway of the structure (see Fig. 1 of the main text), and composed of two superposed and sliding parts:

- the left part of the nomogram contains the ESs of the input to the structure, as well as the (reversed) reference time axis;
- the right part of the nomogram contains the trapezoids as detailed in the Sect. ”The trapezoid method” of the manuscript.

The nomogram necessary to apply the *trapezoid method* to a given problem is implemented in a dynamical manner (the trapezoid set shifts at each crossing step toward the left, using the reference time axis of the parallel input spike as guide), in order to visualize for each step the progression of the parallel input pattern toward the trapezoid set. For each point in time, the sum of the intersections give us the S_p of the target neuron.

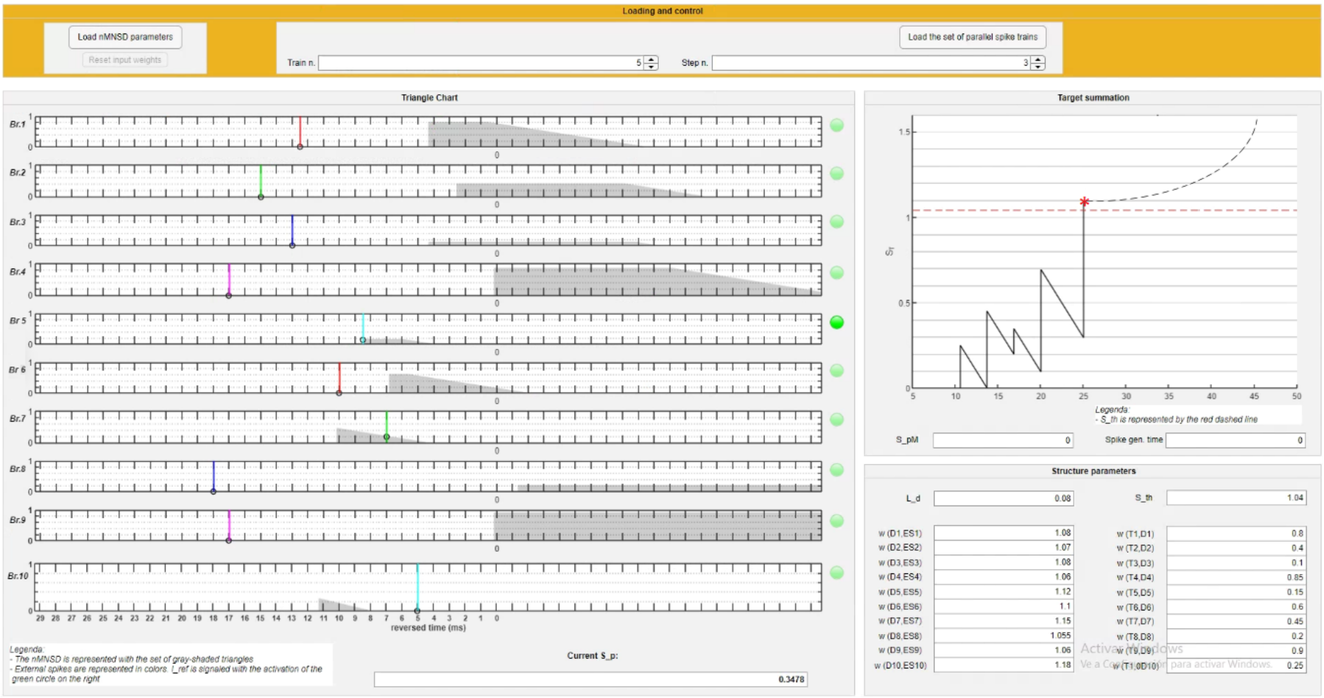

Figure S1. Visualization tool for the trapezoid method. On the left, a 10-branch trapezoid chart is illustrated (green indicators indicate the branch involved in the current crossing step). On top right the corresponding activity at the target neuron is illustrated (*summation peaks* in the time domain). The user can verify the equivalence of the methods looking at the respective S_p values computed.

Appendix 2. Computation of the $\overline{\Delta_{ES}}$ coefficients

Given a fictive reference time c, it is easily possible to define the left exremes of the remaining trapezoids in terms of relative intervals (i.e., the PPT of the structure in terms of preferential relative intervals). Let's consider the nMNSD hit by its PPT (i.e., $\overline{\Delta t_{ES_{i-1,i}}} = \Delta t_{ES_{i-1,i}}$), and ES_1 placed at $t = c$. We note that:

$$\begin{aligned}\overline{\Delta t_{ES_{1,2}}} = \Delta t_{ES_{1,2}} = -\Delta t_{in\, D_{1,2}} = ttf_2 - ttf_1 = \frac{1}{S_{D_2} - 1} - \frac{1}{S_{D_1} - 1} = \\ = \frac{1}{w_{D_2,ES_2} - 1} - \frac{1}{w_{D_1,ES_1} - 1}\end{aligned} \tag{S1}$$

and

$$\begin{aligned}\overline{\Delta t_{ES_{2,3}}} = \Delta t_{ES_{2,3}} = -\Delta t_{in\, D_{2,3}} = ttf_3 - ttf_2 = \frac{1}{S_{D_3} - 1} - \frac{1}{S_{D_2} - 1} = \\ = \frac{1}{w_{D_2,ES_2} - 1} - \frac{1}{w_{D_1,ES_1} - 1}\end{aligned} \tag{S2}$$

in general

$$\begin{aligned}\overline{\Delta t_{ES_{n-1,n}}} = \Delta t_{ES_{n-1,n}} = -\Delta t_{in\, D_{n-1,n}} = ttf_n - ttf_{n-1} = \frac{1}{S_{D_n} - 1} - \frac{1}{S_{D_{n-1}} - 1} = \\ = \frac{1}{w_{D_n,ES_n} - 1} - \frac{1}{w_{D_{n-1},ES_{n-1}} - 1}\end{aligned} \tag{S3}$$

Note that, since:

$$\overline{\Delta t_{ES_{2,3}}} = \Delta t_{ES_{2,3}} = ttf_3 - ttf_2 \tag{S4}$$

we have:

$$\overline{\Delta t_{ES_{1,3}}} = \Delta t_{ES_{1,3}} = \frac{1}{w_{D_3,ES_3} - 1} - \frac{1}{w_{D_1,ES_i} - 1} \tag{S5}$$

and in general:

$$\overline{\Delta t_{ES_{i,j}}} = \Delta t_{ES_{i,j}} = \frac{1}{w_{D_j,ES_j} - 1} - \frac{1}{w_{D_i,ES_i} - 1} \tag{S6}$$

In table S1 we summarize the analytical method for the trapezoid tool.

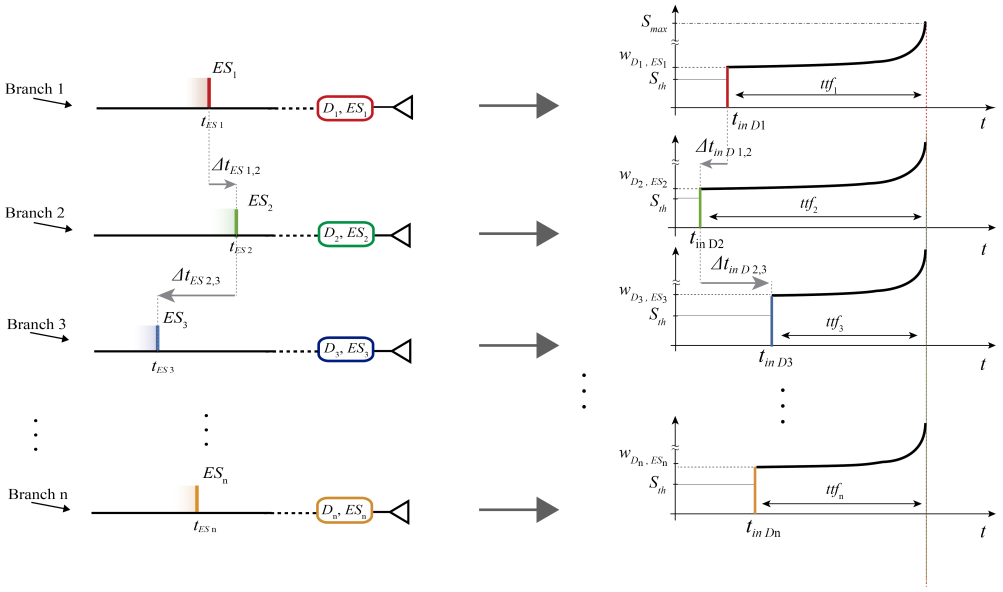

Figure S2. Computation of the $\Delta t_{ES_{i-1,i}}$ coefficients. We hypothesize that the PPT of the nMNSD is entering through input terminals (at left). After being processed by the delay neurons (at right), for definition it complies with the simultaneity condition at the target level.

Table S1. The trapezoid method in four steps.

**Step**	**Procedure**
1. Find the crossing order	Subtract element by element the spike times of the current multi-neuronal input to the left abscissae of the trapezoids:<br>$\langle \overline{v_1}, \overline{v_2}, ..., \overline{v_n} \rangle - \langle t_{ES_1}, t_{ES_2}, ..., t_{ES_n} \rangle$<br>The crossing order is obtained by taking into account the sequence of indices (i.e., by ordering in a descending manner the values of the resulting vector).
2. Anchor the patterns through $\boldsymbol{ref_k}$	For each crossing step compute the S_{p,ref_k} (i.e., the S_p given by the sum of the target efficacies related to the crossing step ref_k). To find the target efficacies for a generic crossing step, align the associated $t_{ES_{ref_k}}$ to the left abscissa of the $ref_k - th$ trapezoid. This can be done by imposing the following condition:<br>$\overline{v_{ref_1}} = t_{ES_{ref_1}}$<br>referring to the equivalence between the expressions 11a and 11b, we obtain:<br>$c = t_{ES_{ref_k}} - \overline{\Delta t_{ES_{1,ref_k}}}$<br>being $\overline{\Delta t_{ES_{1,ref_k}}}$ the relative interval of the PPT between branches 1 and ref_k. We know both the values of the input weights of the structure and ES_{ref_k}, then through the application of Eq. 12, c can be easily found. By using Eq. 12 we also find the values of the preferential relative intervals<br>$\langle \overline{\Delta t_{ES_{1,2}}}, \overline{\Delta t_{ES_{2,3}}}, ..., \overline{\Delta t_{ES_{n-1,n}}} \rangle$.<br>Now, using the equivalence between 11a and 11c, we find the values of the vector of absolute nMNSD left side abscissae anchored to the current input pattern through ref_k. We are now ready to perform the summation for the generic crossing step.
3. Compute the target efficacies $\boldsymbol{e_{ES_{i,ref_k}}}$	Considering the arrival of a new contribution from branch ref_k to the target, to obtain the S_{p,ref_k} the target efficacies of all the branches have to be summed up. The target efficacy of the pulse arriving from the same branch (i.e. $i = ref_k$) is equals to $w_{T,D_{ref_k}}$. The residual contributions of the past arrivals to the target can be identified in the trapezoid chart as the intersections of the other ESs with the upper perimeters of the corresponding trapezoids. They can be computed individually using one of the three following relations:<br>$e_{ES_{i,ref_k}} = w_{T,D_i}$,<br>if $\overline{v_i} - \overline{v_{ref_k}} + t_{ES_{ref_k}} - t_{ES_i} \geq 0$<br>and $\overline{v_i} - \overline{v_{ref_k}} + t_{ES_{ref_k}} - t_{ES_i} < rect_i$<br>$e_{ES_{i,ref_k}} = w_{T,D_i} - L_d \cdot (\overline{v_i} - rect_i - t_{ES_i})$,<br>if $\overline{v_i} - \overline{v_{ref_k}} + t_{ES_{ref_k}} - t_{ES_i} \geq rect_i$<br>and $\overline{v_i} - \overline{v_{ref_k}} + t_{ES_{ref_k}} - t_{ES_i} < rect_i + \frac{w_{T,D_i}}{L_d}$<br>$e_{ES_{i,ref_k}} = 0$,<br>if $t_{ES_i} - \overline{v_i} > rect_i + \frac{w_{T,D_i}}{L_d}$<br>or $\overline{v_i} - \overline{v_{ref_k}} + t_{ES_{ref_k}} - t_{ES_i} < 0$<br>The first equation is used when the previous arrival ES_i crosses the related trapezoid in its *rectangle part*. The second equation is used when the previous arrival ES_i crosses the related trapezoid in its *triangle part*. The third equation is used when the ES_i we are considering is not crossing the related trapezoid because already finished or not yet started.
4. Compute $\boldsymbol{S_{p,ref_k}}$	We can now compute the $S_{p,ref_{k_0}}$ of a generic crossing step ref_{k_0} by summing up all the $e_{i,ref_{k_0}}$ contributions<br>$S_{p,ref_{k_0}} = \sum_{i=1}^{n} e_{i,ref_{k_0}}$<br>We repeat this procedure for each crossing step, obtaining<br>$\langle S_{p,ref_1}, S_{p,ref_2}, ..., S_{p,ref_k}, ..., S_{p,ref_n} \rangle$
